# Anti-trypanosomal screening of Salvadoran flora

**DOI:** 10.1007/s11418-021-01562-6

**Published:** 2021-09-16

**Authors:** Ulises G. Castillo, Ayato Komatsu, Morena L. Martínez, Jenny Menjívar, Marvin J. Núñez, Yoshinori Uekusa, Yuji Narukawa, Fumiyuki Kiuchi, Junko Nakajima-Shimada

**Affiliations:** 1 Laboratorio de Investigación en Productos Naturales, Facultad de Química y Farmacia, Universidad de El Salvador, Final Av. de Mártires y Héroes del 30 de Julio, San Salvador, 1101 El Salvador; 2 Faculty of Pharmacy, Division of Natural Medicines, Keio University, 1-5-30 Shibakoen, Minato-ku, Tokyo, Tokyo 105-8512 Japan; 3 Ministerio de Cultura, Museo de Historia Natural de El Salvador, San Salvador, 1101 El Salvador; 4 Graduate School of Health Sciences, Gunma University, 3-39-22 Showamachi, Maebashi, Gunma 371-8514 Japan

**Keywords:** Chagas disease, *Trypanosoma cruzi*, Salvadoran flora, *Piper jacquemontianum*, Flavonoids

## Abstract

Chagas disease is caused by the protozoan parasite *Trypanosoma cruzi*, and in Central America, it is considered one of the four most infectious diseases. This study aimed to screen the anti-trypanosomal activity of plant species from Salvadoran flora. Plants were selected through literature search for plants ethnobotanically used for antiparasitic and Chagas disease symptomatology, and reported in Museo de Historia Natural de El Salvador (MUHNES) database. *T. cruzi* was incubated for 72 h with 2 different concentrations of methanolic extracts of 38 species, among which four species, *Piper jacquemontianum*, *Piper lacunosum*, *Trichilia havanensis*, and *Peperomia pseudopereskiifolia*, showed the activity (≤ 52.0% viability) at 100 µg/mL. Separation of the methanolic extract of aerial parts from *Piper jacquemontianum* afforded a new flavanone (**4**) and four known compounds, 2,2-dimethyl-6-carboxymethoxychroman-4-one (**1**), 2,2-dimethyl-6-carboxychroman-4-one (**2**), cardamomin (**3**), and pinocembrin (**5**), among which cardamomin exhibited the highest anti-trypanosomal activity (IC_50_ = 66 µM). Detailed analyses of the spectral data revealed that the new compound **4**, named as jaqueflavanone A, was a derivative of pinocembrin having a prenylated benzoate moiety at the 8-position of the A ring.

**Graphic abstract:**

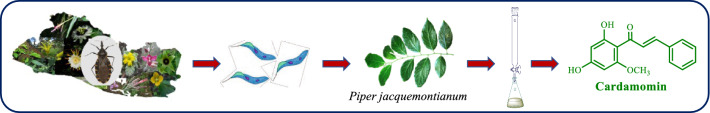

**Supplementary Information:**

The online version contains supplementary material available at 10.1007/s11418-021-01562-6.

## Introduction

Chagas disease is considered the fourth highest burden of infectious diseases in Central America, behind HIV/AIDS, acute respiratory infections, and acute diarrheal diseases [1]. *Trypanosoma cruzi* has been identified as the causal agent of Chagas disease. This protozoan parasite is mainly transmitted through contact with the feces of hematophagous triatomine insects and infects a broad range of mammalian species including humans. In Latin America, Chagas disease continues to be an important public and social health problem, and the World Health Organization [2] considers it as one of the neglected tropical diseases (NTDs).

El Salvador has a population of 6.5 million and approximately 39% of the population is at risk of contracting this disease [3]. The latest official data on Chagas disease, from the Health’s Ministry of El Salvador, conclude that between 2014 and 2017, 771 chronic cases and 53 acute cases were reported and the prevalence of *T. cruzi* in blood banks is among the highest in the Americas, fluctuating between 1.5 and 3.9% in 2008–2016 [1].

At present, Chagas disease is being treated mainly with nifurtimox (NF) and benznidazole (BNZ). Nifurtimox and benznidazole achieve cure rates of 70% and 75%, respectively, in acute cases, and a 100% cure is obtained in congenital cases if the treatment is carried out during the first year of life. However, both drugs are administered for at least 30–60 days and produce side effects in 30% of the cases. Other drugs that have been administered are itraconazole and posaconazole. In acquired chronic cases, a 20% cure and 50% improvement of electrocardiographic changes are obtained with itraconazole [4].

The development of new, safer, and more effective trypanocidal drugs remains a current challenge, as drugs for Chagas disease are not considered a high priority by the R&D-based pharmaceutical industry [5]. Throughout the history of mankind, natural products have been decisive for the discovery of new drugs, since they have enormous structural diversity as compared to conventional synthetic molecules and the screening of these natural sources remains one of the most attractive routes for this purpose [6, 7]. Various plant species have been tested in the search for natural products to combat Chagas disease caused by *T. cruzi*, exhibiting in many cases high trypanocidal activity and low toxicity [8–11].

The present work aimed to assess the in vitro activity of some Salvadoran plant extracts against the epimastigotes forms of *T. cruzi*. Thirty-eight species of plants belonging to 19 genera in 15 families were investigated. Trypanocidal activity was observed in the methanolic extracts obtained from *Peperomia pseudopereskiifolia*, *Piper jacquemontianum*, *P. lacunosum* (Piperaceae), and *Trichilia havanensis* (Meliaceae). Methanolic extract of aerial parts from *P. jacquemontianum* was fractionated, resulting in the isolation and structural elucidation of a new flavanone (**4**), together with four known compounds (**1**–**3** and **5**). Anti-trypanosomal activity of the isolated compounds was evaluated.

## Results and discussion

Bibliographic research for plants ethnobotanically used for antiparasitic and Chagas disease symptomatology (fatigue, depression, constipation, and gastric pains) or heart complaints [12, 13], and reported in Museo de Historia Natural de El Salvador (MUHNES) database, resulted in 350 species. From these species, 97 species belonging to promising botanical families and genera, from which characteristic compounds of proven trypanocidal potential have been reported, were selected as candidates for anti-trypanosomal screening. Among these species, 38 species were collected and used for the screening (Table 1).

**Table 1:** List of Salvadoran plants collected to determine anti-trypanosomal activity

No.	Family	Scientific name^1^	Vernacular name	Part^2^	Voucher number	Collection date/ place^3^
1	Acanthaceae	*Justicia carthagenesis* Jacq.	“Hierba del susto”	A	J. Menjívar et al. 4264	Jul 2018/1
2	Acanthaceae	*Hypoestes phyllostachya* Baker.	“Pecocita”	A	J. Menjívar et al. 4690	May 2018/2
3	Aristolochiaceae	*Aristolochia salvadorensis* Standl.	“Guaco”	A	J. Menjívar et al. 5099	Jun 2018/3
4	Asteraceae	*Baccharis trinervis* Pers.	“Arroz con leche”	A	J. Menjívar et al. 4260	Aug 2018/2
5	Boraginaceae	*Ehretia latifolia* Loisel	Unknown	R and L	J. Menjívar et al. 4633	Aug 2018/4
6	Ebenaceae	*Diospyros salicifolia* Humb. and Bonpl. ex Willd.	Unknown	SB	J. Menjívar et al. 4262	Jul 2018/1
7	Euphorbiaceae	*Acalypha firmula* Müll.Arg.	“Gusanito”	A	J. Menjívar et al. 4622	Aug 2018/2
8	Euphorbiaceae	*Acalypha setosa* A.Rich	“Gusanito”	A	J. Menjívar & M. Núñez 4263	May 2018/5
9	Fabaceae	*Erythrina poeppigiana* (Walp.) O.F.Cook	"Pito extranjero"	L and SB	J. Menjívar et al. 4287	Jul 2018/5
10	Fabaceae	*Lysiloma auritum* (Schltdl.) Benth.	“Quebracho”	SB	J. Menjívar et al. 4659	Sept 2018/6
11	Fabaceae	*Lysiloma divaricatum* Jacq.	“Cicahuite”	SB	J. Menjívar et al. 4660	Sept 2018/6
12	Fabaceae	*Mimosa albida* Humb. and Bonpl. ex Willd.	“Zarza”	A	J. Menjívar et al. 4597	Jul 2018/4
13	Melastomataceae	*Miconia argentea* DC.	“Cirin”	L	J. Menjívar et al. 4261	Aug 2018/2
14	Melastomataceae	*Miconia guatemalensis* Cogn.	“Cirin”	L	J. Menjívar et al. 4697	Jul 2018/4
15	Melastomataceae	*Miconia lauriformis* Naudin	“Cirin”	L	J. Menjívar et al. 4614	Aug 2018/2
16	Meliaceae	*Trichilia havanensis* Jacq.	“Barrehornos”	SB	J. Menjívar et al. 4977	Jul 2018/1
17	Meliaceae	*Trichilia havanensis* Jacq.	“Barrehornos”	SB	J. Menjívar et al. 4221	Aug 2018/2
18	Meliaceae	*Trichilia hirta* L.	“Ceibillo”	SB	J. Menjívar et al. 4665	Sept 2018/6
19	Meliaceae	*Trichilia martiana* C.DC.	“Barrehornos”	SB	J. Menjívar et al. 4227	Aug 2018/2
20	Moraceae	*Dorstenia drakena* L.	“Hierba del sapo”	A	J. Menjívar et al. 4252	Jul 2018/1
21	Lauraceae	*Persea caerulea* (Ruiz and Pav.) Mez	“Aguamico”	SB	J. Menjívar et al. 4288	Oct 2018/7
22	Lauraceae	*Persea schiedeana* C.F.Gaertn.	“Chucte”	L and SB	J. Menjívar et al. 4205	Nov 2018/5
23	Lauraceae	*Persea standleyi* C.K. Allen	“Guacamico”	SB	J. Menjívar et al. 4649	Sept 2018/2
24	Piperaceae	*Peperomia obtusifolia* (L.) A.Dietr.	Unknown	A	J. Menjívar et al. 4599	Jul 2018/4
25	Piperaceae	*Peperomia pseudopereskiifolia* C. DC.	Unknown	A	J. Menjívar et al. 4658	Sept 2018/2
26	Piperaceae	*Peperomia quadrifolia* Miq.	Unknown	A	J. Menjívar et al. 4654	Sept 2018/2
27	Piperaceae	*Piper amalago* L.	Unknown	A	J. Menjívar et al. 4653	Sept 2018/2
28	Piperaceae	*Piper bredemeyeri* J. Jacq.	Unknown	A	J. Menjívar et al. 4621	Aug 2018/2
29	Piperaceae	*Piper jacquemontianum* Kunth	Unknown	A	J. Menjívar et al. 4247	Jun 2018/3
30	Piperaceae	*Piper lacunosum* Kunth	“Cordoncillo”	A	J. Menjívar et al. 4648	Sept 2018/2
31	Piperaceae	*Piper standleyi* Trel.	“Cordoncillo”	A	J. Menjívar et al. 4598	Jul 2018/4
32	Piperaceae	*Piper xanthostachyum* C. DC.	Unknown	A	J. Menjívar et al. 4655	Sept 2018/2
33	Rutaceae	*Zanthoxylum kellermanii*	“Cedro espino”	L and SB	J. Menjívar et al. 4620	Jul 2018/2
34	Sapindaceae	*Exothea paniculate* Radlk.	Unknown	L	J. Menjívar et al. 4660	Sept 2018/2
35	Solanaceae	*Solanum candidum* Lindl.	Unknown	A	J. Menjívar et al. 4615	Aug 2018/2
36	Solanaceae	*Solanum lanceolatum* Cav.	“Cuerno de vaca”	A	J. Menjívar et al. 4624	Aug 2018/4
37	Solanaceae	*Solanum myriacanthum* Dunal	“Huevos de gato”	A	J. Menjívar et al. 4616	Aug 2018/2
38	Solanaceae	*Solanum torvum* Sw.	Unknown	A	J. Menjívar et al. 4618	Aug 2018/2

^1^Scientific names are given following international plant name index (IPNI)

^2^*A* arial parts; *L* leaves; *SB* stem bark; *R* roots

^3^Collection place number: 1: PNA Bosque de Cinquera; 2: NP Montecristo; 3: NP El Imposible; 4: NP Complejo Los Volcanes; 5: Cantón El Jocotón; 6: Potonico; 7: Chinchontepec Volcano

*PNA* protected natural area; *NP* national park

**Fig. 1:**
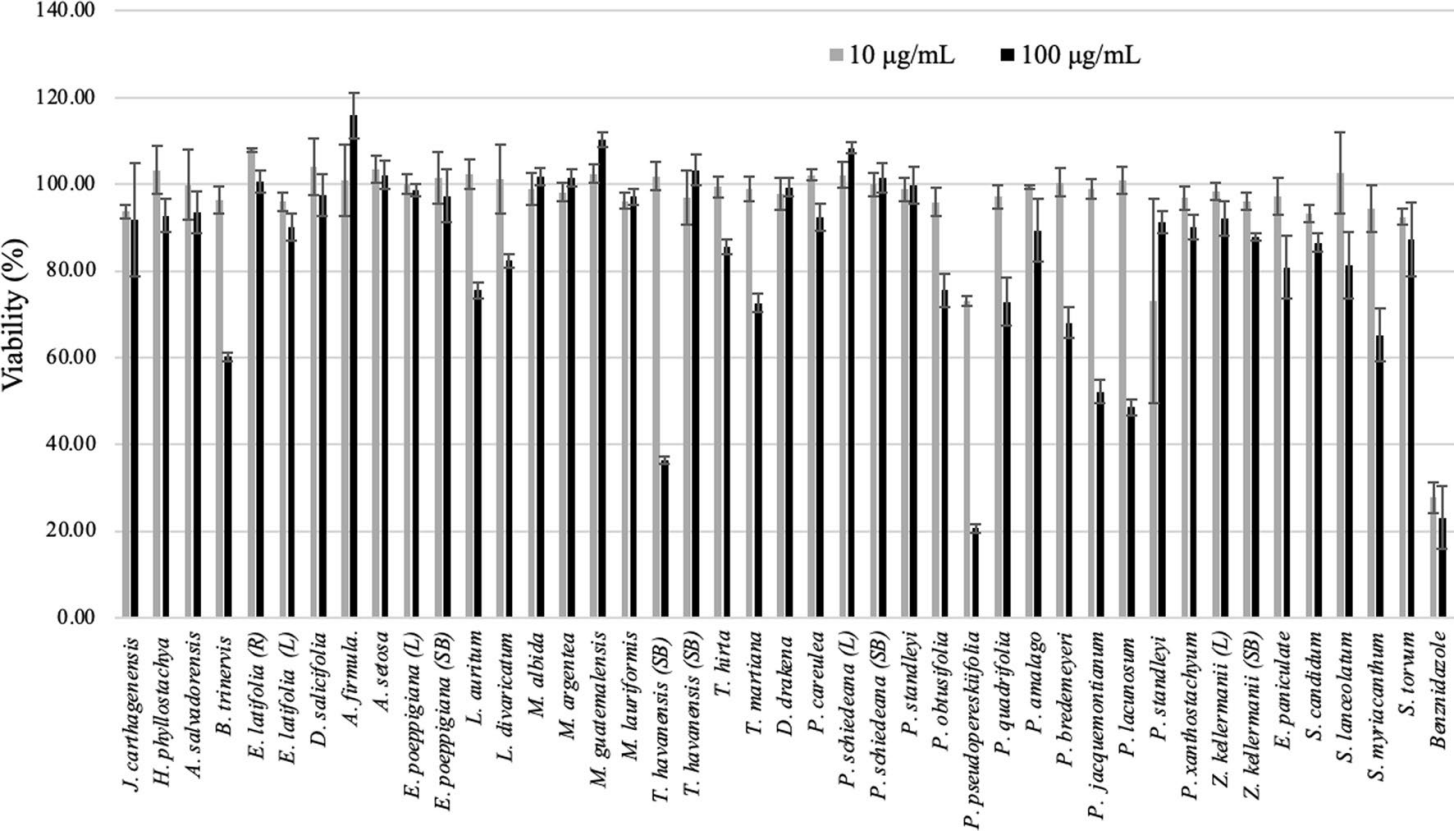
Anti-trypanosomal activity of Salvadoran flora

To carry out the anti-trypanosomal screening, MeOH extracts of collected plants were prepared. The yields of the extract ranged between 5.8–18.1%, 2.7–11.5%, 10.4–48.8%, and 6.3% for aerial parts, leaves, stem bark, and roots, respectively. The activity of 38 plant species from El Salvador against *T. cruzi* is summarized in Fig. 1 (also see S3). Although there are no widely accepted criteria to consider a promising extract or compound [13], taking into account the criteria of Osorio et al. [14], an extract is considered moderately active if it reduces viablility of *T. cruzi* around 50% at 100 µg/mL. Among the 38 plant species, four species, *Peperomia pseudopereskiifolia, Piper jacquemontianum, P. lacunosum* (Piperaceae)*,* and *Trichilia havanensis* (Meliaceae), showed moderate activity at 100 µg/mL.

The Piperaceae family includes a large number of plants used in tropical and subtropical regions. The *Piper* and *Peperomia* genera are the most representative of this botanical family and *Piper* species are commonly used in Latino-American traditional medicines for the treatment of protozoal diseases [15]. From the Piperaceae family, some species have been tested against *T. cruzi* and some bioactive compounds have been isolated such as flavonoids, terpenoids, lignans, chromanes, and alkaloids [13, 15, 16]. Concerning genus *Peperomia*, the main classes of compounds described are, phenylpropanoids, lignans, pyrones, aliphatic and aromatic amides, alkaloids, polyketides, benzoic acid derivatives, and chromenes, among which trypanocidal activity was reported for lignans and benzoic acid derivatives [17, 18].

Genus *Trichilia* has been studied for different biological activities, including trypanocidal activity [19, 20]. In a study of Meliaceae and Rutaceae family, the branches of *T. ramalhoi* were one of the most active extracts [21]. According to Pizzolatti et al. [19], the bark of *T. catigua* showed activity against trypomastigotes of *T. cruzi.* From *T. havanensis* some metabolites have been isolated, such as triterpenoids, tetranortriterpenoid, hydroxybutenolide derivative, and limonoids [22–24]. In a study carried out against *Trypanosoma brucei*, the bark extract of *T. emetica* showed activity attributed to the presence of limonoids [20].

In this study, we isolated the constituents of *P. jacquemontianum*. This plant is a shrub 1–4 m in high, and native to Central America and the Caribbean lowlands [25]. In various Latin American countries, *P. jacquemontianum* is used in folklore medicine to treat skin ailments, infections, anemia, and body aches [26, 27]. In Panama, it is traditionally used as a remedy for fever, headache, and cold, nervousness, diabetes, stomachache [28]. Chemical study of this species has been mainly performed on essential oils. Linalool and *E*-nerolidol were reported as the major constituents of its essential oil [29, 30], whereas a recent study revealed that the composition was variable and only *E*-nerolidol was detected as a common constituent among the eight cultivars from different places in Guatemala [31]. Although linalool has been reported to show anti-trypanosomal activity [32], nothing has been reported on anti-trypanosomal constituents of the MeOH extract of this species.

Aerial parts of *P. jaquemontianum* (510 g) were extracted with MeOH at room temperature to give 83.5 g of the extract. As a preliminary experiment, the MeOH extract (1.0 g) was separated by a silica gel column chromatography with stepwise gradients of hexane and ethyl acetate and then MeOH to obtain nine fractions (D1–D9), among which D3–D6 inhibited the growth of epimastigotes of *T. cruzi* more than 50% at 100 mg/mL. The major constituents of the active fractions were isolated to obtain compounds **1** (58 mg) and **2** (8.6 mg) from D4 and D6, respectively.

For further isolation of the constituents, the MeOH extract (24.1 g) was separated by silica gel column chromatography (hexane-AcOEt and then AcOEt-MeOH) to give nine fractions (E1–E9). Fractions E1, E4, E6, and E2, E3, E5 inhibited the growth of the epimastigotes more than 75% and 50%, respectively, at 100 mg/mL. From the major active fraction E4, compound **5** (6.3 mg) was obtained by silica gel column chromatography (hexane-AcOEt) and preparative HPLC (ODS, 75% MeOH). Separation of the other major fraction E5 by ODS (MeOH-H_2_O) and silica gel (hexane-AcOEt) column chromatography and preparative HPLC (CH_3_CN-H_2_O) to give compounds **3** (47 mg) and **4** (2.4 mg). Compounds **2** and **1** were identified as 2,2-dimethyl-6-carboxychroman-4-one [30] and its methyl ester [29, 33], respectively, by comparison of their spectral data with those reported in the literature. It is the first time that these compounds were isolated from aerial parts of *P. jacquemontianum*.

**Fig. 2:**
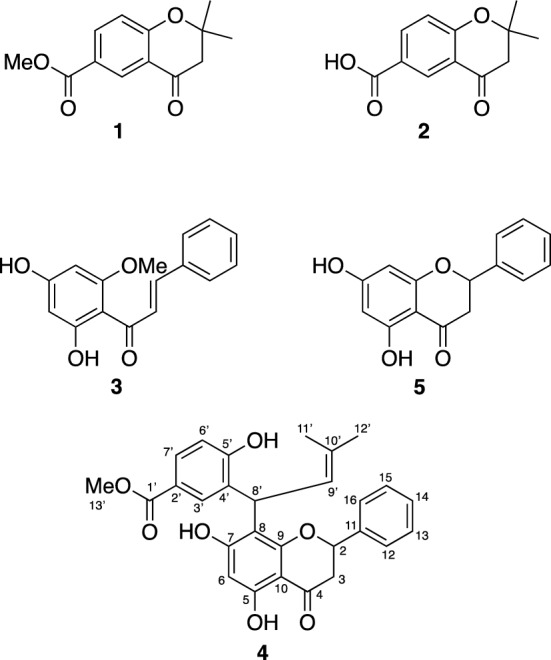
Structures of isolated compounds from *P. jaquemontianum*

Compounds **3** and **5** were also identified as cardamomin [34] and pinocembrin [35–37], respectively (Fig. 2). As compound **5** did not show significant optical rotation, it seems to be a racemic mixture. Pinocembrin and cardamomin have previously been isolated from *Piper* species [38–40].

**Fig. 3:**
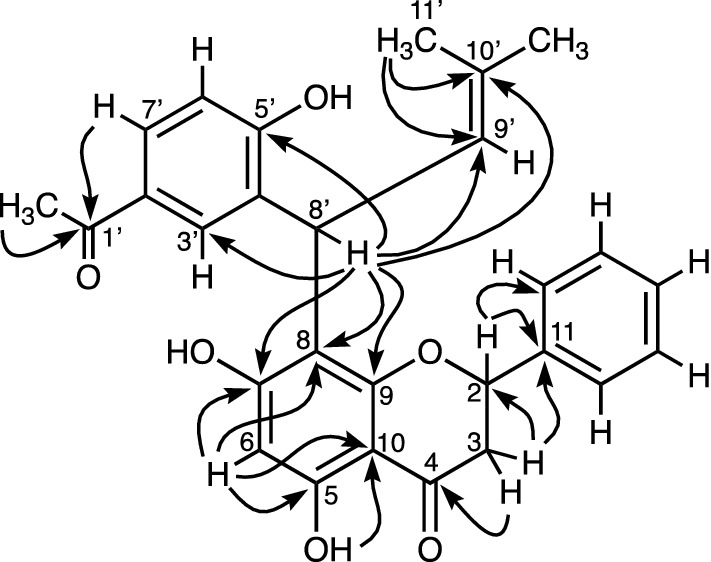
Selected key HMBC correlations of compound **4**

The new compound **4** was obtained as white powder. Its ^1^H NMR spectrum (Table 2) showed similar signals [δ: 12.2 (1H, s, OH), 7.44 (3H, overlapped, H-13, H-14, H-15), 7.39 (2H, br d, *J* = 7.3 Hz, H-12, H-16), 5.45 (1H, br d, *J* = 13.2 Hz, H-2), 3.09 (1H, dd, *J* = 13.2, 17.5 Hz, H-3), 2.84 (1H, dd, *J* = 2.0, 17.5 Hz, H-3)] with those of pinocembrin (**5**) except that only a singlet [6.07 (1H, br s, H-6)] was observed for ring A, indicating that compound **4** is a derivative of pinocembrin (**5**) having a substituent on the A ring. The spectrum also showed signals of a tri-substituted benzene ring [8.01 (1H, br s, H-3’), 7.81 (1H, br d, *J* = 8.3 Hz, H-7’), 6.83 (1H, d, *J* = 8.3 Hz, H-6’)], a methoxy group [3.85 (3H, s, H-13’)], and a prenyl group [1.79 (3H, s, H-12’), 1.68 (3H, s, H-11’), 5.98 (1H, br d, *J* = 8.2 Hz, H-9’), 5.39 (1H, d, *J* = 8.2 Hz, H-8’)]. These signals, together with the ^13^C NMR signals and the HMQC correlations (Table 2), suggested that the substituent on the A ring is a prenylated methyl benzoate derivative. The connectivity of these groups was concluded from the HMBC correlations as shown in Fig. 3. The HMBC spectrum also showed correlations from the methine proton [5.39 (1H, d, *J* = 8.2 Hz, H-8’)] of the prenyl group to C-7, C-8 and C-9 carbons of the ring A of pinocembrin moiety (Fig. 3), confirming the substitution position of this group at the 8-position on the A ring. Thus, the structure of compound **4** was concluded as shown in Fig. 2. Flavanones and prenylated benzoic acid derivatives [30, 35, 41–43] have been reported from various *Piper* species. However, a compound having both of these moieties has not been reported. As this is a new compound, it is named as jaqueflavanone A. In the HPLC purification of this compound, the presence of a closely related compound was observed. The ^1^H NMR spectrum of a mixture of this compound and compound **4** suggested that it is a diastereomer of compound **4**. However, this compound could not be obtained as a single compound. Compound **4** has two asymmetric carbons (C-2 and C-8’). As pinocembrin isolated from this extract was a racemic mixture, the stereochemistry of C-2 seems racemic. However, the stereochemistry of the other carbon could not be determined.

**Table 2:** NMR data of compound **4** in CDCl_3_

Position	13C	1H	HMBC
2	79.9	g: 5.45, 1H, br d (13.2)	d, k1
3	43.4	c: 2.84, 1H, dd (2.0, 17.5) d: 3.09, 1H, dd (13.2, 17.5)	
4	196.2		c, d
5	162.5		i, n
6	97.7	i: 6.07, 1H, br s	n
7	162.7		f, i
8	108.8		a, b, f, i
9	159.6		f
10	103.5		i, n
11	138.0		d, g, k2
12	126.1	k1: 7.39, 1H, br d (7.3)	g, k2
13	128.9	k2: 7.44, 1H, overlapped	k1
14	126.0	k2: 7.44, 1H, overlapped	k1
15	128.9	k2: 7.44, 1H, overlapped	k1
16	126.1	k1: 7.39, 1H, br d (7.3)	g, k2
1’	167.5		e, l, m
2’	128.5		f, j
3’	131.3	m: 8.01, 1H, br s	f, l
4’	122.1		j, m
5’	158.2		f, j, l, m
6’	116.0	j: 6.83, 1H, d (8.3)	
7’	129.7	l: 7.81, 1H, br d (8.3)	m, f
8’	33.6	f: 5.39, 1H, d (8.2)	j, h, m
9’	122.9	h: 5.98, 1H, br d (8.2)	a, b, f
10’	136.1		a, b, f
11’	18.2	a: 1.68, 3H, s	b, h
12’	25.9	b: 1.79, 3H, s	a, h
13’	52.0	e: 3.85, 3H, s	
5-OH	–	n: 12.2, 1H, s

The IC_50_ values of the isolated compounds against epimastigotes of *T. cruzi* were 1.28 mM (**1**), > 2 mM (**2**), 66 µM (**3**), 100 µM (**4**), and 714 µM (**5**). Cardamomin (**3**) has been reported to show trypanocidal activity against *Trypanosoma brucei* [44]. Synthesis and anti-trypanosomal activity of chalcone derivatives [45, 46] and chalcone-based compounds [47–49] have been reported.

## Conclusion

In the present study, in vitro anti-trypanosomal activities of thirty-eight species from Salvadoran flora are reported. The most active methanolic extracts were from *Peperomia speudopereskiifolia* (Piperaceae), *Trichilia havanensis* (Meliaceae), *Piper lacunosum* (Piperaceae), and *Piper jacquemontianum* (Piperaceae). These results confirmed the effectiveness of the ethnobotanically used plants. The activity-guided fractionation of *P. jacquemontianum* resulted in the isolation of anti-trypanosomal compounds including cardamomin, whose activity against *Trypanosoma* has been reported. Constituents of the other active species are now under investigation.

Considering these findings, we can conclude that Salvadoran flora is a potential source of anti-trypanosomal substances and that the most promising extracts are potential sources of compounds for the development of more effective drugs for the treatment of Chagas disease. Translation of some in vitro results into in vivo follow-up studies is recommended in the future.

## Materials and methods

### Apparatus

ESI-TOFMS spectra were obtained with a JMS-T100LP AccuTOF LC-plus (JEOL, Tokyo, Japan) operated in negative ion mode. NMR spectra were recorded on an AVANCE 500 spectrometer (Bruker, MA, USA). Tetramethylsilane (TMS) was used as an internal standard. For HPLC profile analysis of crude extracts, a chromatographic system consisted of an LC-2010A HT (Shimadzu Co., Kyoto, Japan) liquid chromatography module equipped with SPD-M30A PDA detector set at 190–700 nm was used. The data were collected and processed using LabSolutions system (Shimadzu Co., Kyoto, Japan). Separation was performed in a column CAPCELL PAK C18 (TYPE: MGII, 2.0 × 150 mm, 5 mm, Shiseido, Tokyo, Japan) at 40ºC. The mobile phase consisted of water (solvent A) and acetonitrile (solvent B), both containing 0.1% (v/v) of formic acid, with a flow rate of 0.2 mL/min. Linear gradient time program was set as follows: 0 min, 15% B; 0–3 min, 15% B; 3–37 min, 100% B; 37–42 min, 100% B. For preparative HPLC, an HPLC system (Shimadzu Co., Kyoto, Japan) equipped with a LC-10ADvp HPLC pump and an SPD-10Avp UV/Vis detector was used. HPLC conditions for the separation are as follows: column, CAPCELL PAK C18 (10 mm i.d. × 150 mm, 5 mm; Shiseido, Tokyo, Japan); mobile phase, 50% MeCN (0 min)–60% MeCN (30 min) (gradient elution, condition 1), 75% MeOH (isocratic elution, condition 2); flow rate, 2.5 mL/min; UV detection, 254 nm.

### Plant selection process

Bibliographic research was done to look for Salvadoran plants with ethnobotanical uses for antiparasitic and Chagas disease symptomatology (fatigue, depression, constipation, gastric pains) or heart complaints [12, 13], resulting in 380 possible plant species. The selection was limited to the plant species reported in Museo de Historia Natural de El Salvador (MUHNES) database, resulting in 350 species. These species were investigated in scientific journals database to find promising botanical families and genera from which bioactive compounds have been reported, moreover, the plants are known to contain characteristic compounds of proven trypanocidal potential. Ninety-seven species resulted as a possible anti-trypanosomal species.

### Plant materials

From the 97 possible species, only thirty-eight species were possible to collect for anti-trypanosomal screening. They were collected under the permission and resolution code MARN-DEV-GVS-040-2018 at protected natural areas of El Salvador in 2018 and identified by Jenny Elizabeth Menjívar Cruz, Curator of the Herbarium at the Museo de Historia Natural de El Salvador (Table 1). A voucher specimen has been deposited for each species in the Herbarium at the MUHNES.

To carried out the bioassay-guided isolation, the aerial parts of *Piper jacquemontianum* Kunth were collected at El Imposible National Park, Ahuachapán, El Salvador (latitude:13°49′49” N, longitude: 89°56′33” W, elevation: 816 m) in June 2019 and the respective voucher (J. Menjivar et al. 4247) specimen has been deposited in the herbarium at MUHNES.

### Preparation of plant extracts

The collected plants were dried at 40 °C for 48–72 h in a circulating air oven (BIOBASE, China, model BOV-V225F) and milled to obtain a particle size ≤ 2 mm (Bel-Art products, USA, model micro-mill). Twenty grams of each sample were extracted with methanol (200 mL × 2) in a magnetic stirrer ultrasonic bath (VWR, USA, model 97,043–988, operating frequency at 35 kHz) for 90 min at 25 °C. Each extract was concentrated under reduced pressure at 40 °C (model RE801, Yamato Scientific Co., Ltd., Japan) to obtain the MeOH extracts.

### Sample preparation for HPLC profile of crude extracts

Each extract (10.0 mg) was dissolved in 1 mL of methanol, using an ultrasonic bath (VWR, model 97,043–988, operating frequency at 35 kHz) at room temperature. A solution of 1 mg/mL was prepared for each extract solution using methanol. The sample solution was filtrated through a 0.45 µm membrane filter before being subjected to HPLC analysis. HPLC profiles of the extracts are shown in the supplemental materials (Figure S7).

### Anti-trypanosomal screening of crude extracts (MTT method)

Ninety-five microliters of epimastigotes (3 × 10^6^ epimastigotes/mL, Tulahuen strain) suspended in GIT medium supplemented with hemin (12.4 µM) were added in each well of 96-well plate. These were incubated at 28 °C for 72 h, after the addition of 5 mL of extracts (100 and 10 µg/mL in DMSO). Benznidazole was used as a positive control. After the incubation time, 10 µL of MTT reagent (5 mg/mL in PBS) was added to each well and incubated for another 24 h. The medium was discarded and the precipitates of formazan were dissolved with 100 µL of DMSO. The measurement of absorbance was performed at 530 nm.

### Anti-trypanosomal test for fractions and isolated compounds (luminescence method)

Epimastigotes of *T. cruzi* (1 × 10^5^ parasites/well) in GIT medium (50 µL) and a sample dissolved in DMSO (0.5 mL) were added to each well of a 384-well white plate (Falcon, 353,988). The plate was incubated at 27 °C for 24 h. After the incubation, 20 µL of CellTiter-Glo reagent (Promega, G7570) were added to each well and the intensity of luminescence (500–670 nm) was measured by a luminometer (Infinite M200 Pro, Tecan). To compensate for the luminescence of the sample itself, each sample solution in GIT medium without epimastigotes was used as a blank. Tamoxifen was used as a positive control (IC_50_ 19.3 mM). To determine IC_50_ values, six different concentrations of each sample were prepared.

### Extraction and isolation of *Piper jacquemontianum*

The dried and ground aerial parts of *Piper jacquemontianum* (510.0 g) were extracted with MeOH (3 × 5 L, 14 days each) at room temperature, and concentrated using a rotary evaporator, yielding a crude extract (83.46 g). A part of the MeOH extract of *P. jaquemontianum* (1.0 g) was subjected to silica gel column chromatography (4 × 15 cm) and successively eluted with hexane: AcOEt = 100:0, 90:10, 70:30, 50:50, 30:70, 10:90, 0:100, then AcOEt: MeOH = 50:50, 0:100 to give fractions D1 (6.7 mg), D2 (30.6 mg), D3 (20.4 mg), D4 (129.7 mg), D5 (50.5 mg), D6 (68.5 mg), D7 (27.6 mg), D8 (221.1 mg), and D9 (158.8 mg). A part of fraction D4 (115 mg) was fractionated by silica gel column chromatography (3 × 25 cm) with hexane: AcOEt = 19: 1 to give D4-1 (0.3 mg), D4-2 (58.4 mg), D4-3 (10.2 mg), D4-4 (52.6 mg), D4-5 (3.2 mg), D4-6 (4.0 mg), D4-7 (5.4 mg), D4-8 (6.3 mg), and D4-9 (15.7 mg). Fraction D4-2 was crystallized from hexane–EtOH to give 2,2-dimethyl-6-carbomethoxychroman-4-one (**1**). Fraction D6 (34.0 mg) was separated by ODS column chromatography (1.5 × 15 cm) with H_2_O: MeOH = 1:1, and then 100% MeOH to give fractions D6-1 (4.6 mg), D6-2 (8.6 mg), D6-3 (0.3 mg), D6-4 (1.2 mg), D6-5 (1.9 mg), and D6-6 (3.0 mg). Fraction D6-2 was crystallized from hexane–EtOH to give 2,2-dimethyl-6-carboxychroman-4-one (**2**).

A part of the MeOH extract of *P. jaquemontianum* (24.1 g) was fractionated by silica gel column chromatography (6 × 22 cm) and successively eluted with hexane: AcOEt = 100:0, 90:10, 70:30, 50:50, 30:70, 10:90, 0:100, and AcOEt: MeOH = 50:50, 0:100 to give 9 fractions: E1 (107.2 mg), E2 (930.0 mg), E3 (3.1 g), E4 (2.1 g), E5 (2.1 g), E6 (819.5 mg), E7 (621.3 mg), E8 (4.5 g), and E9 (4.0 g). The major fraction E4 (2.1 g) was separated by silica gel column chromatography (4 × 15 cm) with hexane: AcOEt = 4:1, 2:1, and then 100% MeOH to give fractions E4-1 (5.7 mg), E4-2 (11.8 mg), E4-3 (601.0 mg), E4-4 (70.6 mg), E4-5 (136.5 mg), E4-6 (206.6 mg), E4-7 (194.0 mg), and E4-8 (565.2 mg). Fraction E4-5 (14.2 mg) was purified by HPLC (HPLC condition 2) to give fractions E4-5–1 (6.3 mg) and E4-5–2 (5.0 mg). Fraction E4-5–1 was crystallized from MeOH to give pinocembrin (**5**). Fraction E5 (2.1 g) was subjected to an ODS column chromatography (4 × 15 cm) and eluted with H_2_O: MeOH = 1: 1, then 100% MeOH to give fractions E5-1 (116.6 mg), E5-2 (665.5 mg), E5-3 (404.7 mg), E5-4 (122.2 mg), E5-5 (231.2 mg), E5-6 (39.0 mg), and E5-7 (289.9 mg). Fraction E5-2 was identified as 2,2-dimethyl-6-carboxychroman-4-one (**2**). Fraction E5-4 was fractionated by silica gel column chromatography (3 × 20 cm) with hexane: AcOEt = 2:1, and then 100% MeOH to give fractions E5-4–1 (47.3 mg), E5-4–2 (12.4 mg), and E5-4–3 (19.3 mg). Fraction E5-4–1 was crystallized from MeOH to give cardamomin (**3**). Fraction E5-4–2 (10.8 mg) was separated by preparative HPLC (HPLC condition 1) to give compound **4** (2.4 mg).

**Jaqueflavanone A** (**4**): white powder, mp 133–135˚C; HR-ESI-TOFMS (neg.) *m*/*z* 473.1592 [M−H] ^−^ (calcd. for C_28_H_25_O_7_, 473.1600); ^1^H NMR (500 MHz, CDCl_3_) δ: 12.2 (1H, s, OH), 8.01 (1H, br s, H-3’), 7.81 (1H, br d, *J* = 8.3 Hz, H-7’), 7.44 (3H, overlapped, H-13/15, H-14), 7.39 (2H, br d, *J* = 7.3 Hz, H-12/16), 6.83 (1H, d, *J* = 8.3 Hz, H-6’), 6.07 (1H, br s, H-6), 5.98 (1H, br d, *J* = 8.2 Hz, H-9’), 5.45 (1H, br d, *J* = 13.2 Hz, H-2), 5.39 (1H, d, *J* = 8.2 Hz, H-8’), 3.85 (3H, s, H-13’), 3.09 (1H, dd, *J* = 13.2, 17.5 Hz, H-3), 2.84 (1H, dd, *J* = 2.0, 17.5 Hz, H-3), 1.79 (3H, s, H-12’), 1.68 (3H, s, H-11’). ^13^C NMR (125 MHz, CDCl_3_) δ: 196.2 (C-4), 167.5 (C-1’), 162.7 (C-7), 162.5 (C-5), 159.6 (C-9), 158.2 (C-5’), 138.0 (C-11), 136.1 (C-10’), 131.3 (C-3’), 129.7 (C-7’), 128.9 (C-13), 128.5 (C-2’), 126.1 (C-12), 126.0 (C-14), 122.9 (C-9’), 122.1 (C-4’), 116.0 (C-6’), 108.8 (C-8), 103.5 (C-10), 97.7 (C-6), 79.9 (C-2), 52.0 (C-13’), 43.4 (C-3), 33.6 (C-8’), 25.9 (C-12’), 18.2 (C-11’).

## Supplementary Information

Below is the link to the electronic supplementary material.

Supplementary file1 (PDF 3565 kb)
