# Supplementary material for: Anti-trypanosomal screening of Salvadoran flora
Source: J Nat Med. 2021 Sep 16;76(1):259–67. doi: 10.1007/s11418-021-01562-6 (PMC8732892; doi:10.1007/s11418-021-01562-6)

Supplementary Materials

Anti-trypanosome screening of Salvadoran flora

Ulises G. Castillo[1], Ayato Komatsu[2], Morena L. Martínez[1], Jenny Menjívar[3], Marvin J. Núñez[1], Yoshinori Uekusa[2], Yuji Narukawa[2], Fumiyuki Kiuchi[2], Junko Nakajima-Shimada[4]

[1]*Laboratorio de Investigación en Productos Naturales, Facultad de Química y Farmacia, Universidad de El Salvador, Final Av. de Mártires y Héroes del 30 de Julio, San Salvador 1101, El Salvador.*

[2]*Division of Natural Medicines, Faculty of Pharmacy, Keio University, 1-5-30 Shibakoen, Minato-ku, Tokyo 105-8512, Tokyo, Japan.*

[3]*Museo de Historia Natural de El Salvador, Ministerio de Cultura, San Salvador 1101, El Salvador.*

[4]*Graduate School of Health Sciences, Gumma University, 3-39-22 Showamachi, Maebashi, 371-8514, Gumma, Japan*

Table of contents

S3. Anti-trypanosomal activity of Salvadoran flora

S4. Figures 4–45 HPLC profile of the MeOH extracts

S25. Spectral data of known compounds

S27. NMR spectra of compound **4** ($CDCl_3$)

S3. Anti-trypanosomal activity of Salvadoran flora

		Viability (%)	
No.	Species	10 µg/mL	100 µg/mL
1	*Justicia carthagenensis* (A)	93.7 ± 1.5	91.8 ± 13.2
2	*Hypoestes phyllostachya* (A)	103.3 ± 5.5	92.8 ± 3.8
3	*Aristolochia salvadorensis* (A)	99.9 ± 8.1	93.6 ± 4.5
4	*Baccharis trinervis* (A)	96.4 ± 3.0	60.1 ± 0.9
5A	*Ehretia latifolia* (R)	107.9 ± 0.4	100.7 ± 2.6
5B	*E. latifolia* (L)	95.9 ± 2.2	90.1 ± 3.0
6	*Diospyros salicifolia* (SB)	104.0 ± 6.5	97.5 ± 4.9
7	*Acalypha firmula* (A)	101.0 ± 8.2	115.8 ± 5.4
8	*A. setosa* (A)	103.5 ± 3.2	102.1 ± 3.2
9A	*Erythrina poeppigiana* (L)	100.1±6.0	98.7±6.1
9B	*E. poeppigiana* (SB)	101.5±3.5	97.3±1.9
10	*Lysiloma auritum* (SB)	102.3 ± 2.2	75.5 ± 1.5
11	*L. divaricatum* (SB)	101.2 ± 7.8	82.3 ± 1.5
12	*Mimosa albida* (A)	98.9 ± 3.8	101.8 ± 2.1
13	*Miconia argentea* (L)	98.2 ± 2.0	101.6 ± 2.0
14	*M. guatemalensis* (L)	102.4 ± 2.0	110.2 ± 1.6
15	*M. lauriformis* (L)	96.2 ± 1.8	97.1 ± 1.8
16	*Trichilia havanensis* (SB)	101.8 ± 3.3	36.5 ± 0.8
17	*T. havanensis* (SB)	96.9±2.5	103.3±1.8
18	*T. hirta* (SB)	99.4 ± 6.2	85.6 ± 3.5
19	*T. martiana* (SB)	99.0 ± 2.9	72.6 ± 2.2
20	*Dorstenia drakena* (A)	97.7 ± 3.6	99.2 ± 2.1
21	*Persea careulea* (SB)	102.1 ± 1.2	92.4 ± 3.1
22A	*P. schiedeana* (L)	102.1 ± 3.0	108.3 ± 1.3
22B	*P. schiedeana* (SB)	99.9 ± 2.8	101.5 ± 3.5
23	*P. standleyi (SB)*	98.8 ± 2.7	99.8 ± 4.13
24	*Peperomia obtusifolia* (A)	95.9 ± 3.3	75.5 ± 3.8
25	*P. pseudopereskiifolia* (A)	73.0 ± 1.2	20.6 ± 1.0
26	*P. quadrifolia* (A)	97.1 ± 2.7	72.8 ± 5.6
27	*Piper amalago* (A)	99.8 ± 0.5	89.3 ± 7.2
28	*P. bredemeyeri* (A)	100.4 ± 3.3	68.0 ± 3.6
29	*P. jacquemontianum* (A)	99.0 ± 2.3	52.0 ± 2.7
30	*P. lacunosum* (A)	100.9 ± 3.2	48.5 ± 1.9
31	*P. standleyi* (A)	73.1 ± 23.6	91.2 ± 2.6
32	*P. xanthostachyum* (A)	96.8 ± 2.7	90.1 ± 2.9
33A	*Zanthoxylum kellermanii* (L)	98.3 ± 1.9	92.2 ± 4.0
33B	*Z. kellermanii* (SB)	96.2 ± 2.0	87.8 ± 0.9
34	*Exothea paniculate* (L)	97.2 ± 4.2	80.8 ± 7.2
35	*Solanum candidum* (A)	93.2 ± 1.9	86.5 ± 2.2
36	*S. lanceolatum* (A)	102.6 ± 9.5	81.3 ± 7.6
37	*S. myriacanthum* (A)	94.5 ± 5.4	65.2 ± 6.7
38	*S. torvum* (A)	92.5 ± 1.7	87.2 ± 8.5
	Benznidazole	27.7 ± 3.5	23.1 ± 7.2

A: aerial parts; L: leaves; SB: stem bark; R: roots. Mean value ± standard deviation (n=3)

S4. Figures 4–45 HPL profile of the MeOH extracts

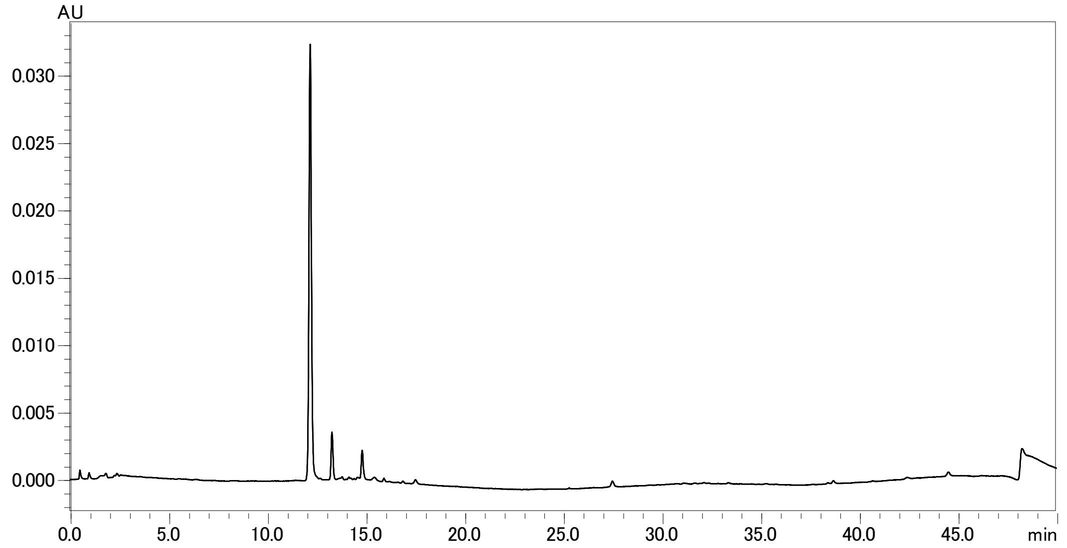

Figure 4. HPLC chromatogram (UV 280 nm) of the extract 1.

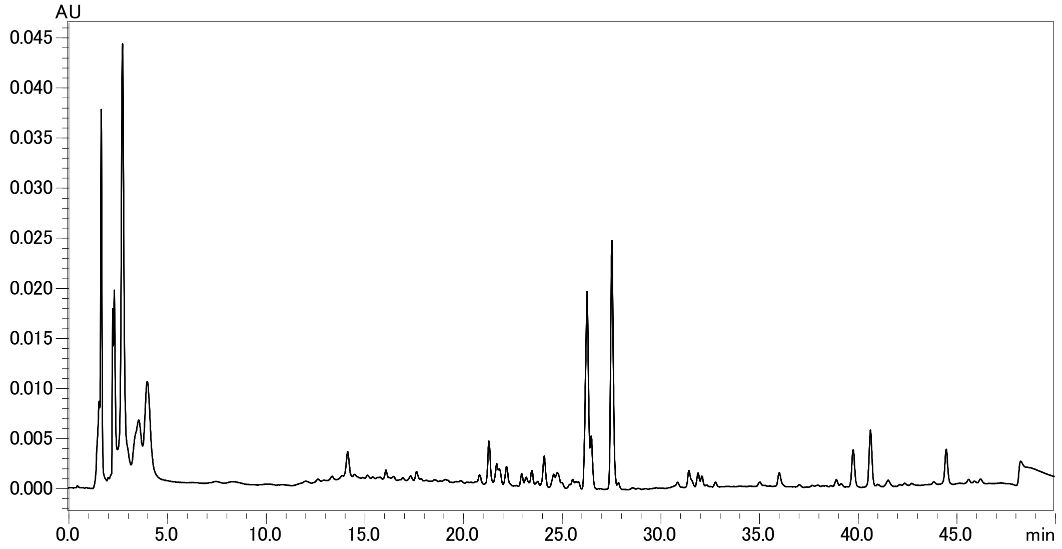

Figure 5. HPLC chromatogram (UV 280 nm) of the extract 2.

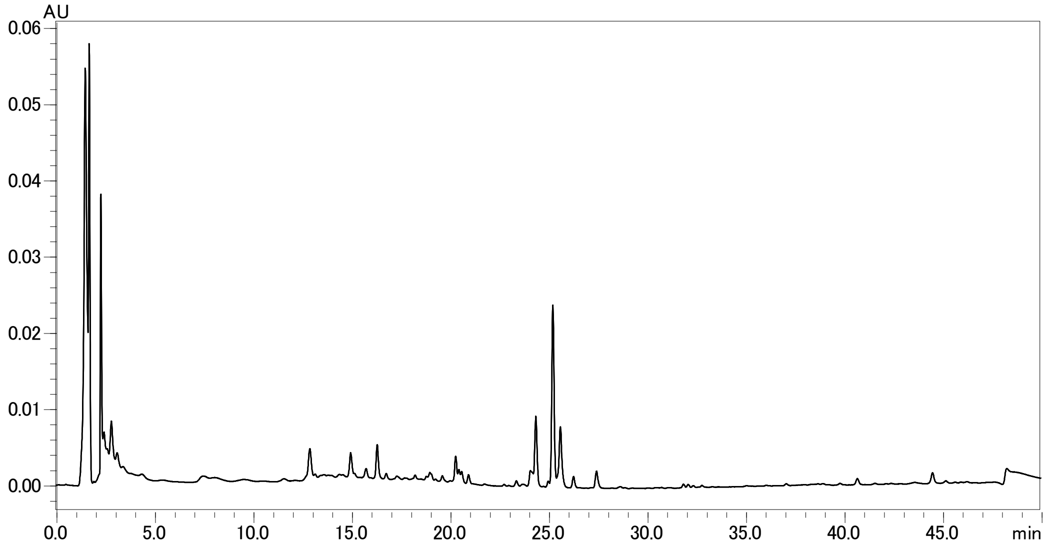

Figure 6. HPLC chromatogram (UV 280 nm) of the extract 3.

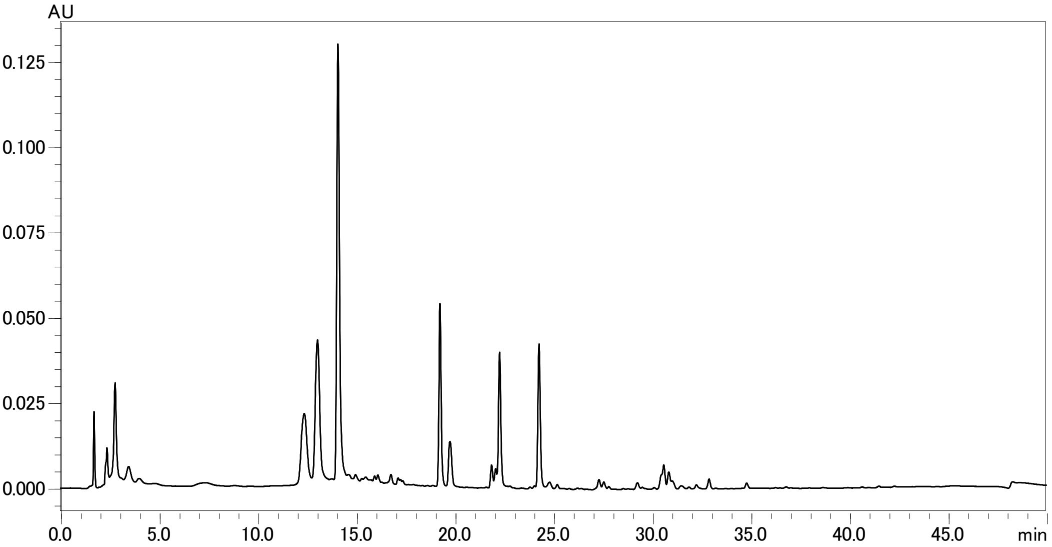

Figure 7. HPLC chromatogram (UV 280 nm) of the extract 4.

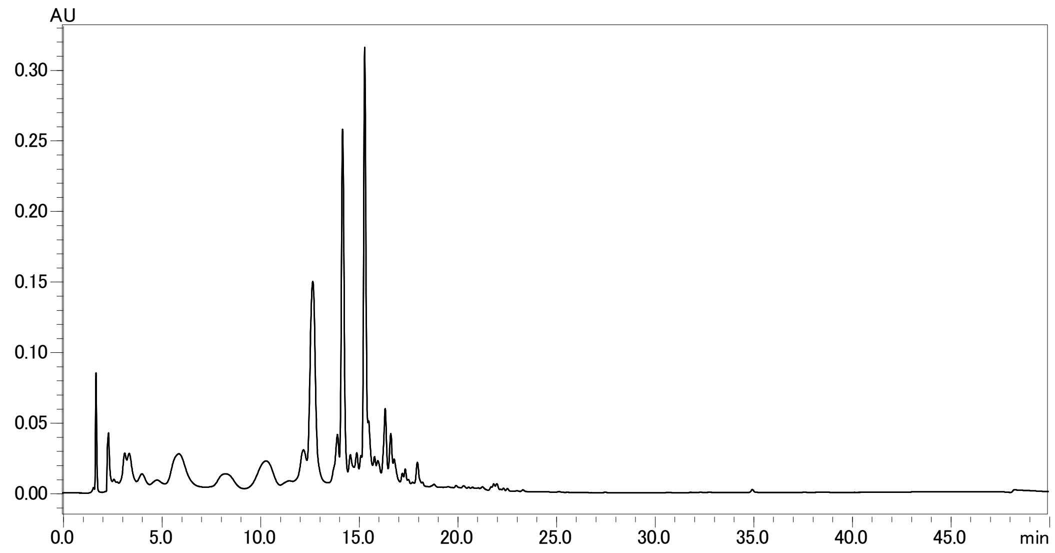

Figure 8. HPLC chromatogram (UV 280 nm) of the extract 5A.

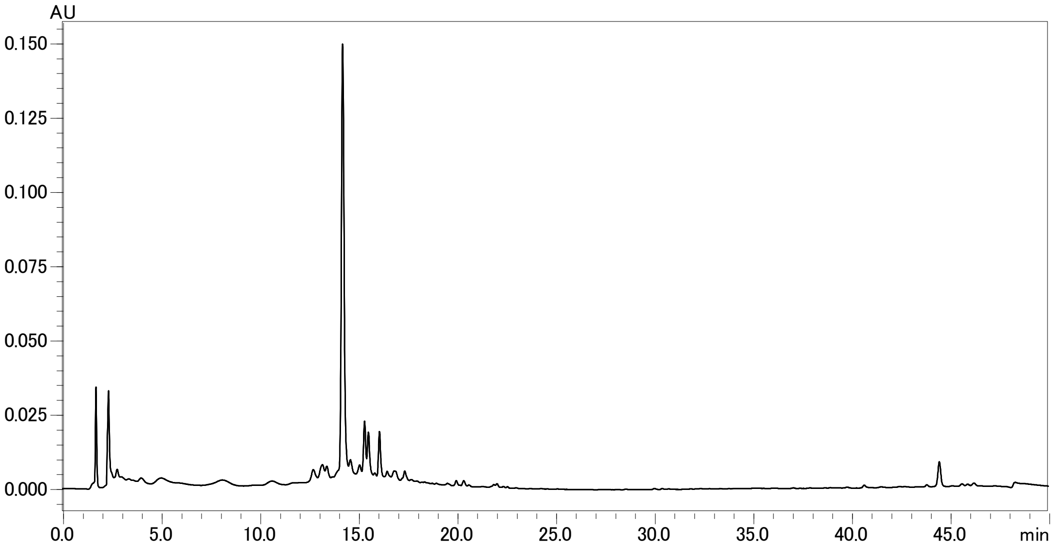

Figure 9. HPLC chromatogram (UV 280 nm) of the extract 5B.

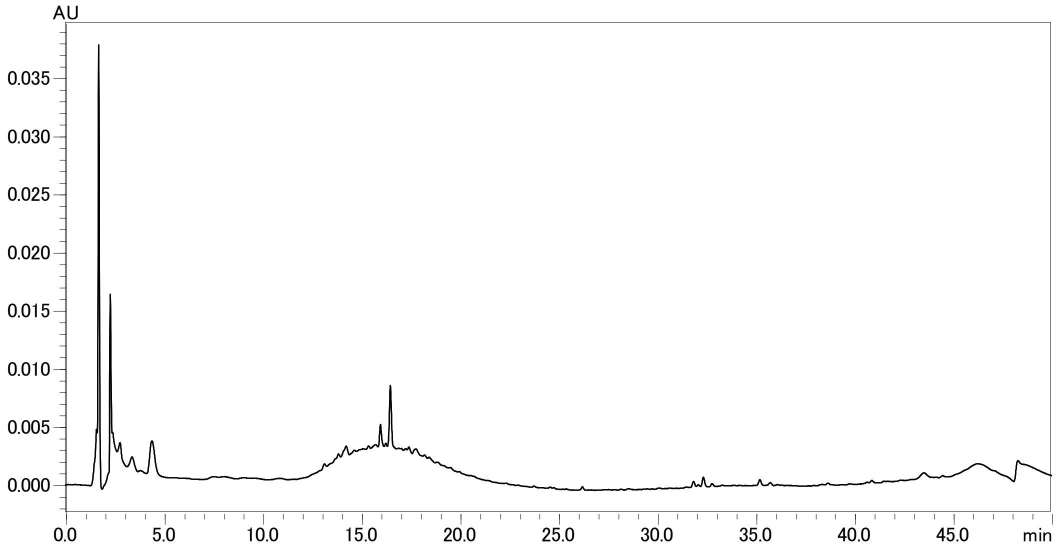

Figure 10. HPLC chromatogram (UV 280 nm) of the extract 6.

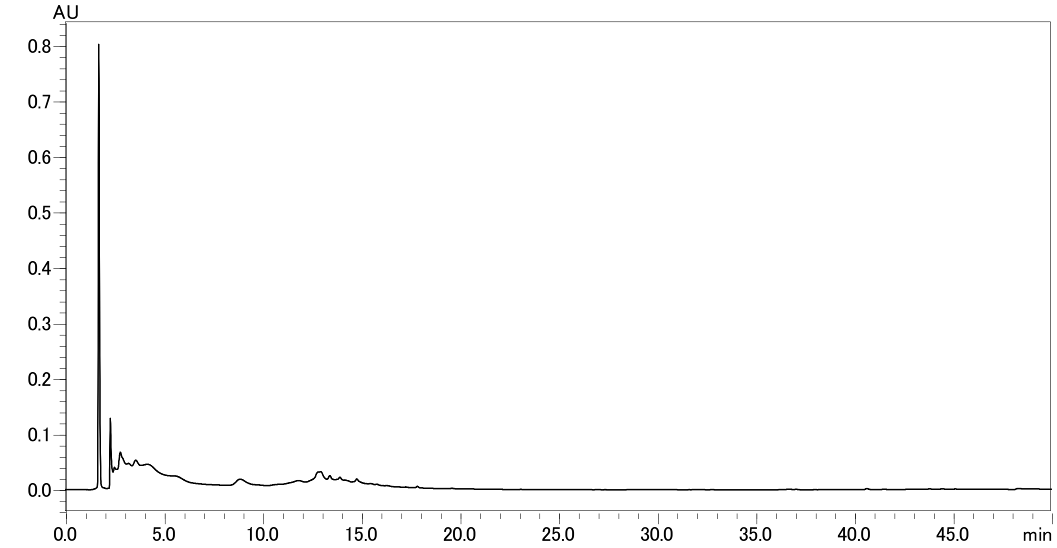

Figure 11. HPLC chromatogram (UV 280 nm) of the extract 7.

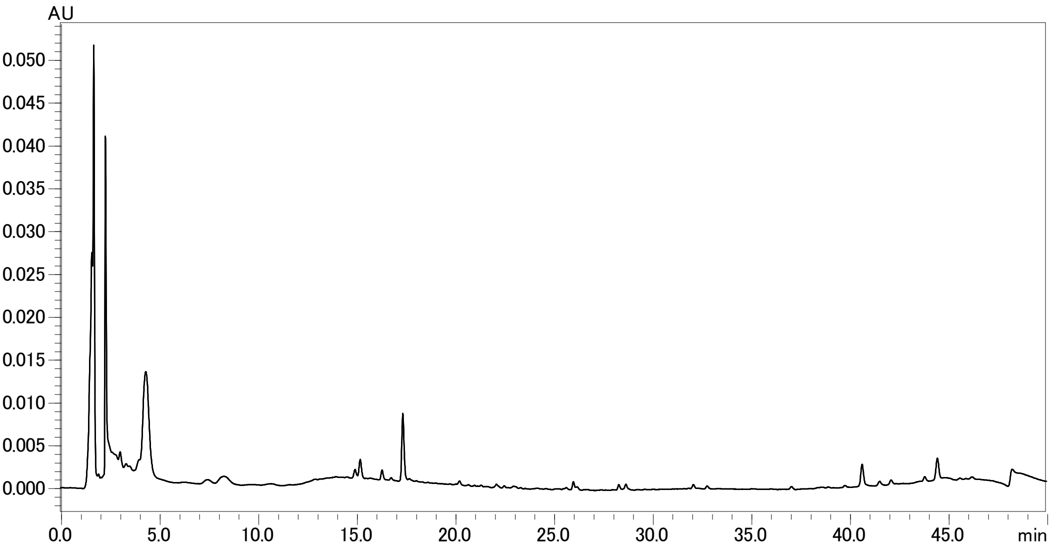

Figure 12. HPLC chromatogram (UV 280 nm) of the extract 8.

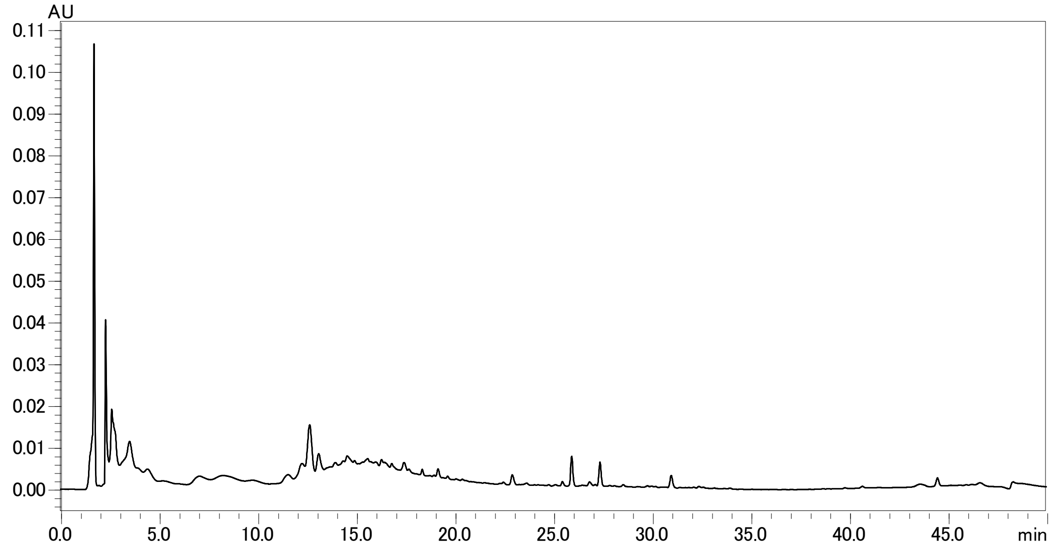

Figure 13. HPLC chromatogram (UV 280 nm) of the extract 9A.

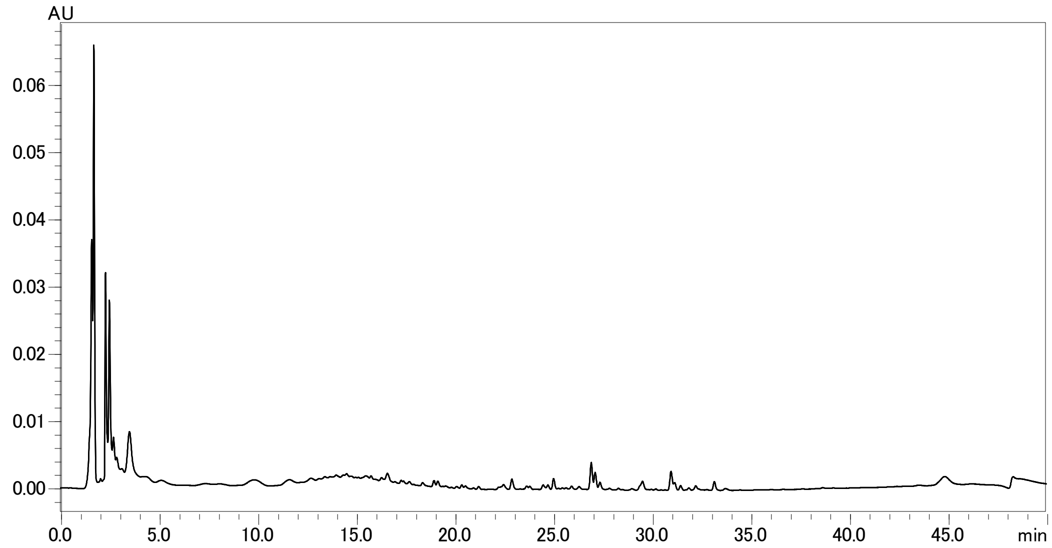

Figure 14. HPLC chromatogram (UV 280 nm) of the extract 9B.

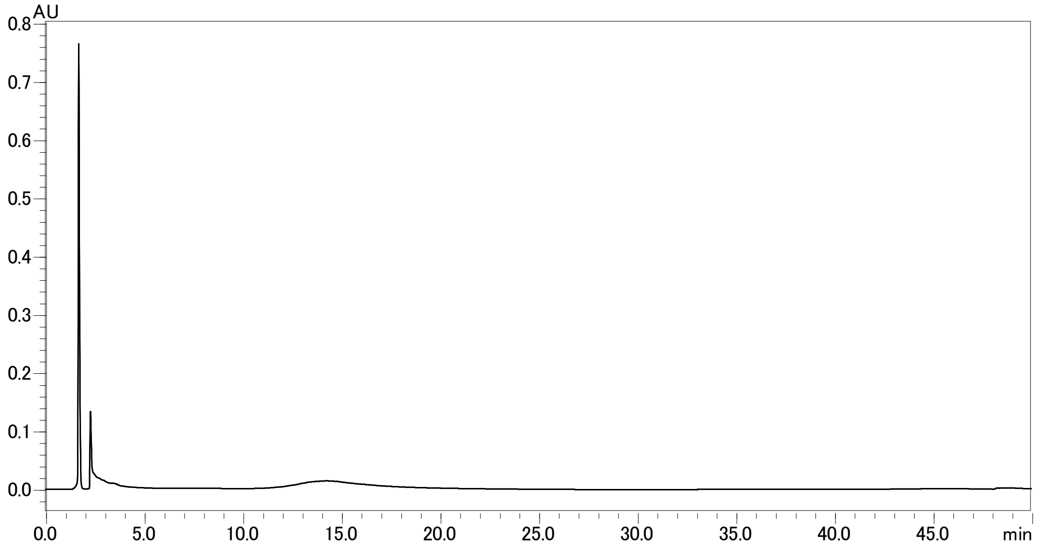

Figure 15. HPLC chromatogram (UV 280 nm) of the extract 10.

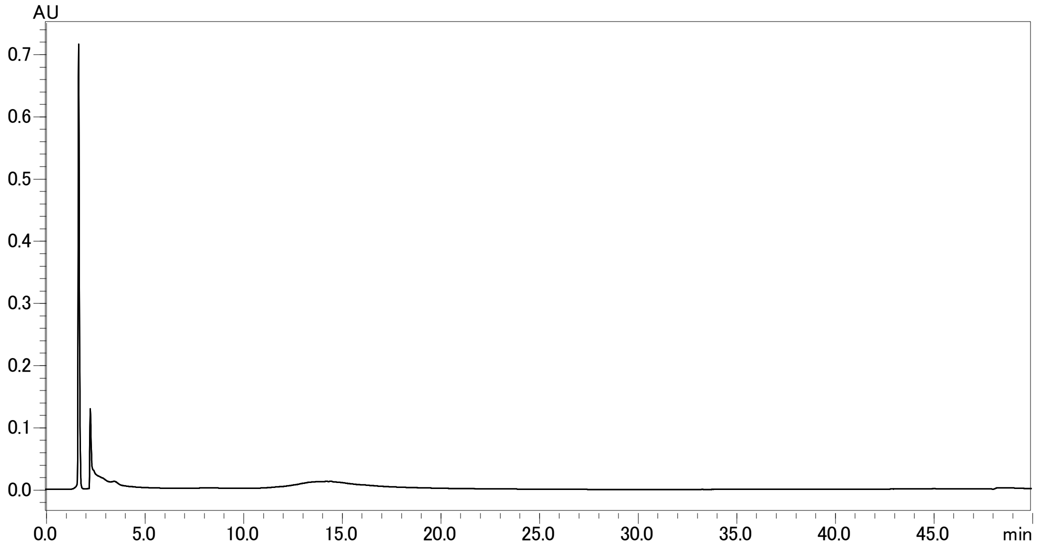

Figure 16. HPLC chromatogram (UV 280 nm) of the extract 11.

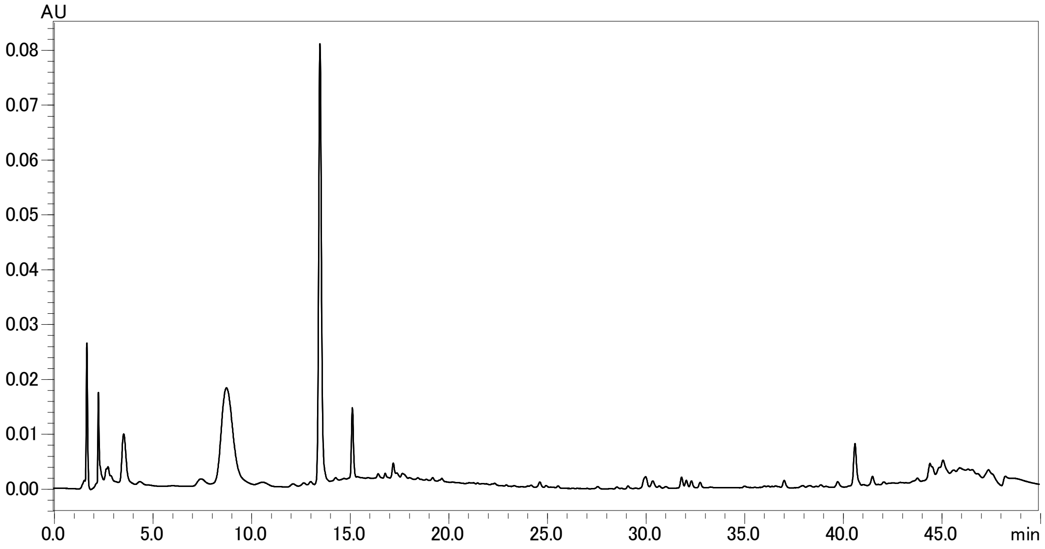

Figure 17. HPLC chromatogram (UV 280 nm) of the extract 12.

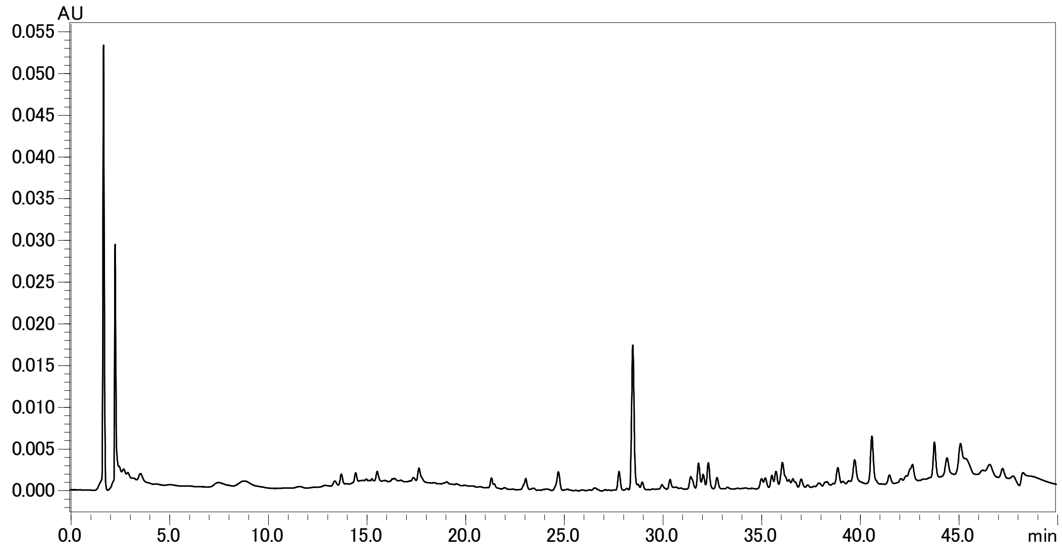

Figure 18. HPLC chromatogram (UV 280 nm) of the extract 13.

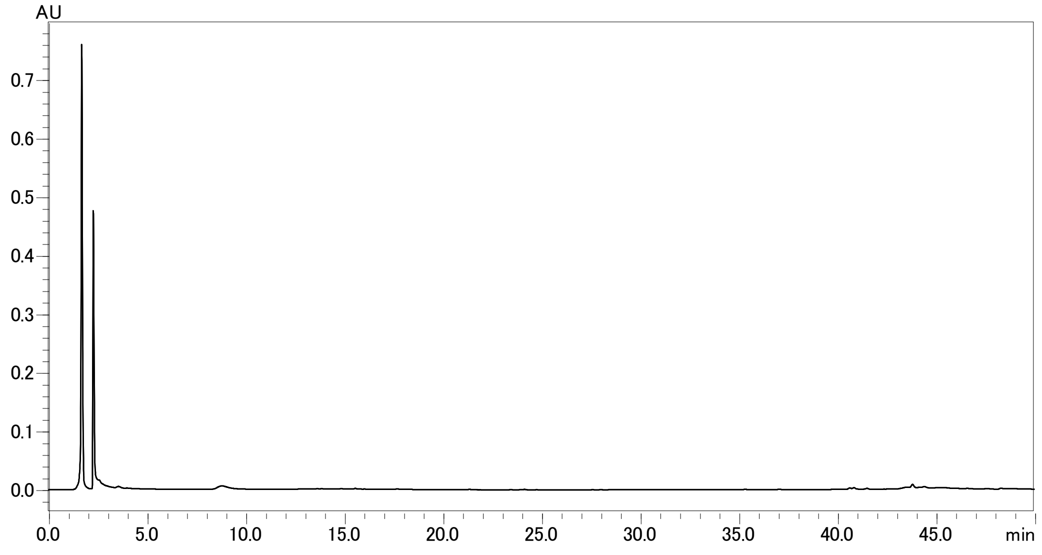

Figure 19. HPLC chromatogram (UV 280 nm) of the extract 14.

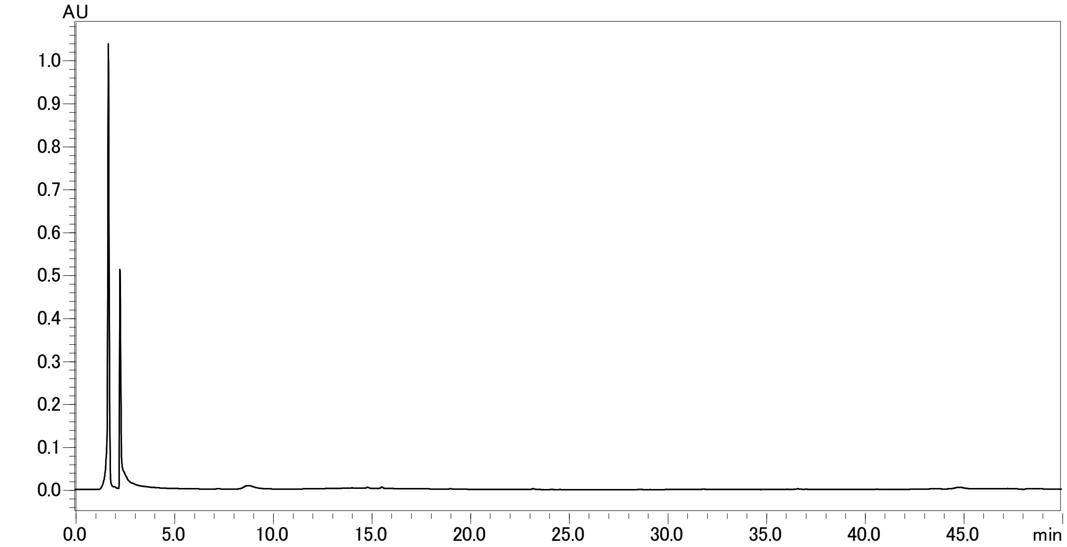

Figure 20. HPLC chromatogram (UV 280 nm) of the extract 15.

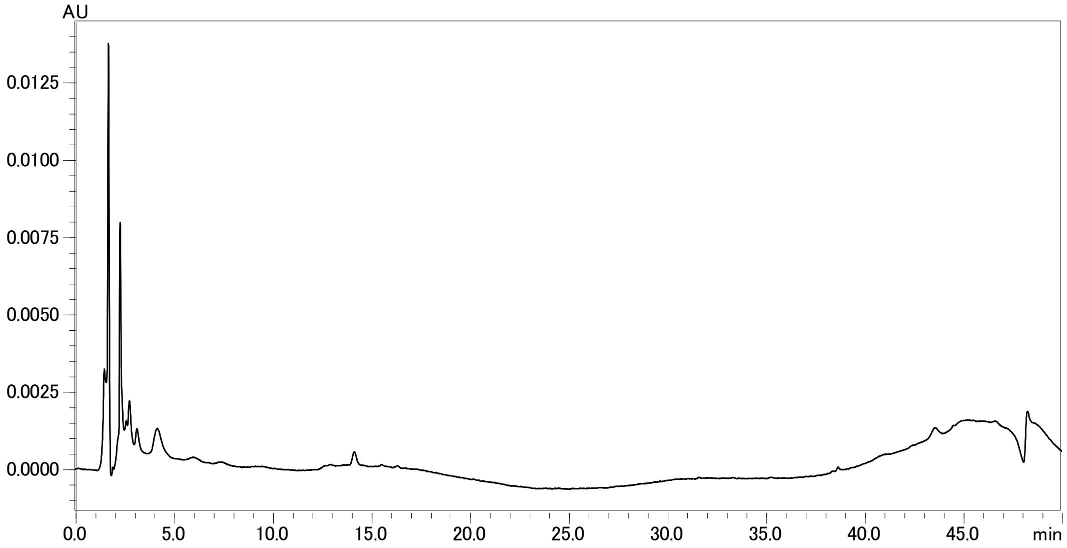

Figure 21. HPLC chromatogram (UV 280 nm) of the extract 16.

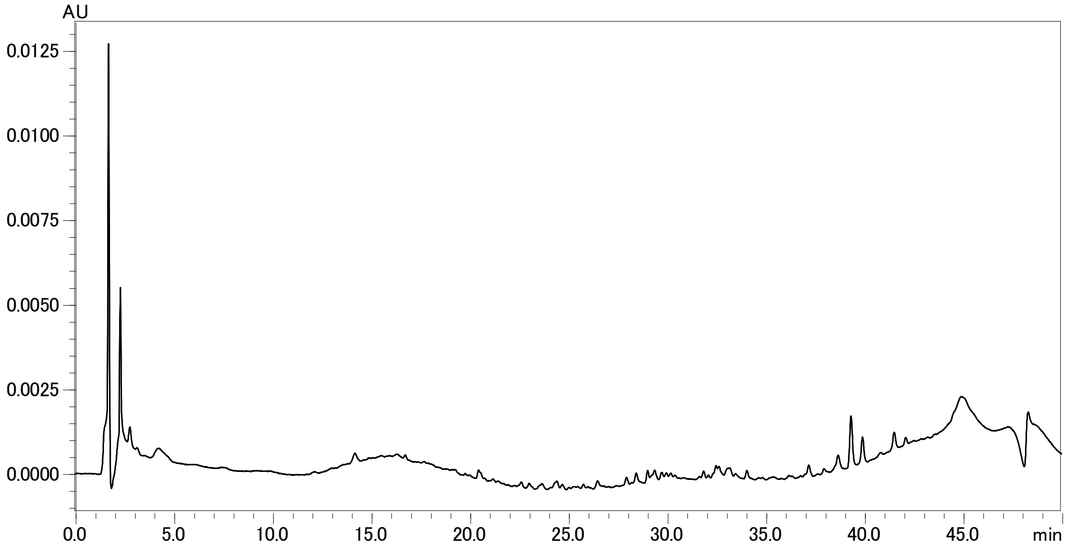

Figure 22. HPLC chromatogram (UV 280 nm) of the extract 17.

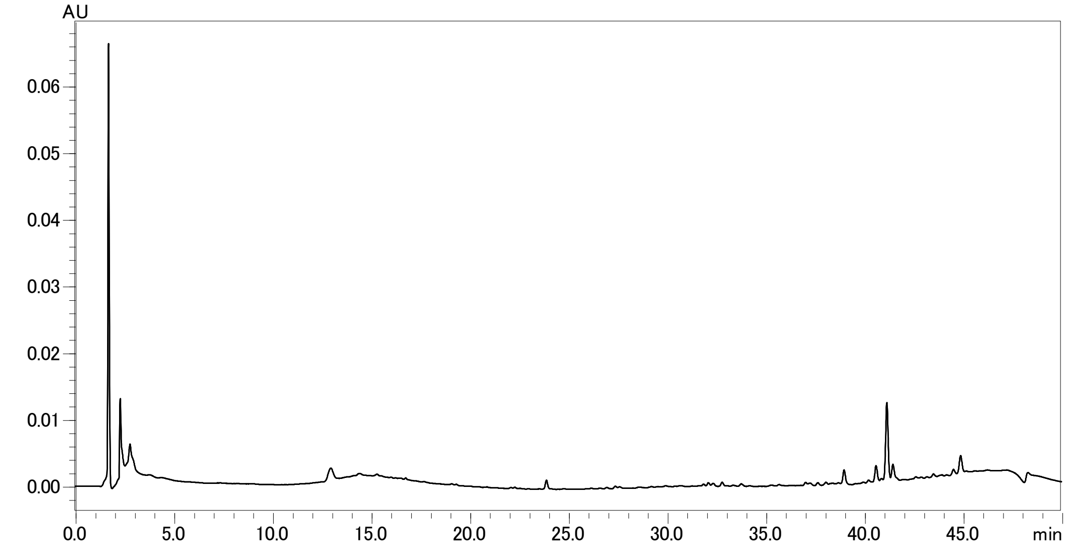

Figure 23. HPLC chromatogram (UV 280 nm) of the extract 18.

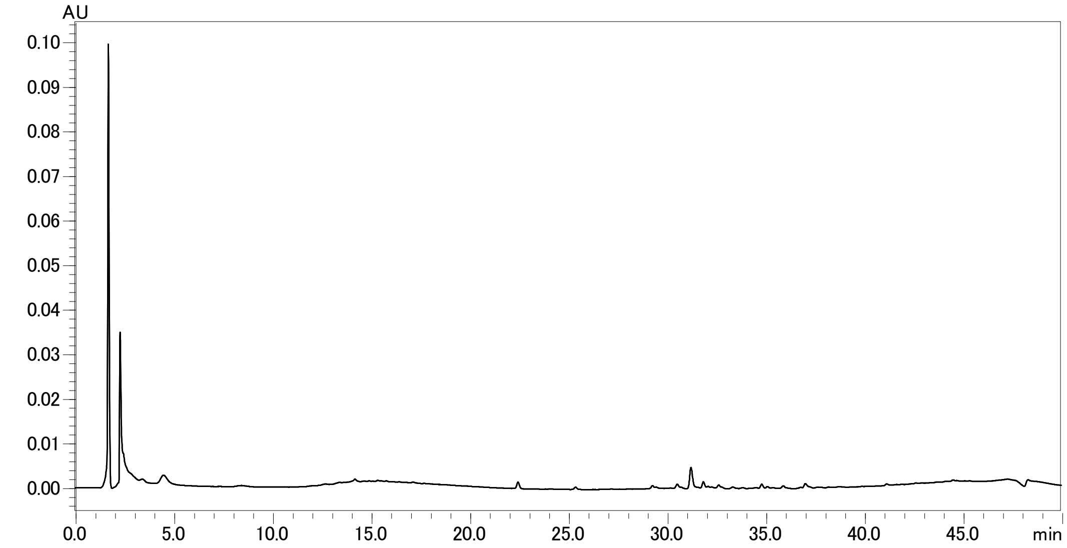

Figure 24. HPLC chromatogram (UV 280 nm) of the extract 19.

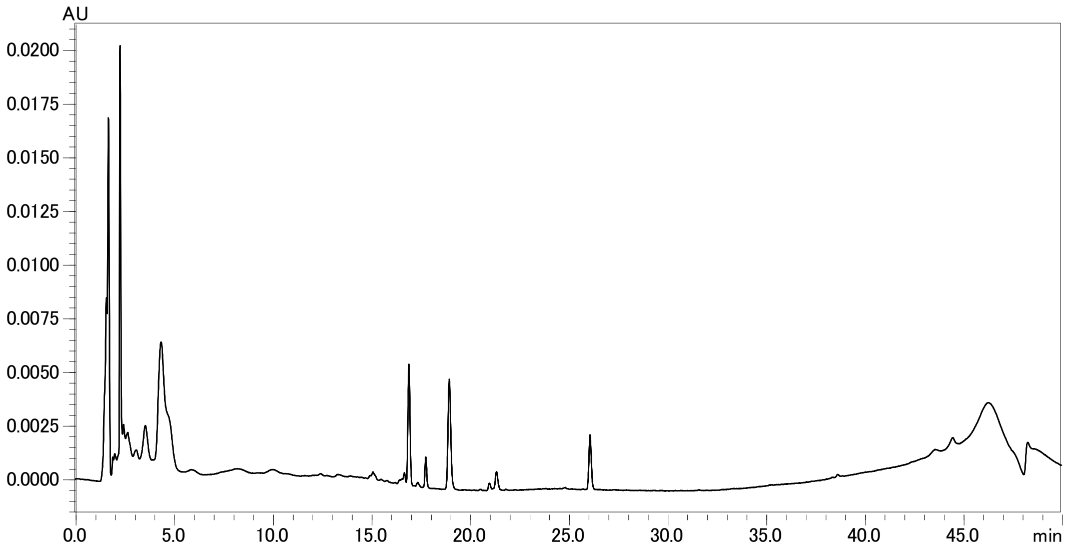

Figure 25. HPLC chromatogram (UV 280 nm) of the extract 20.

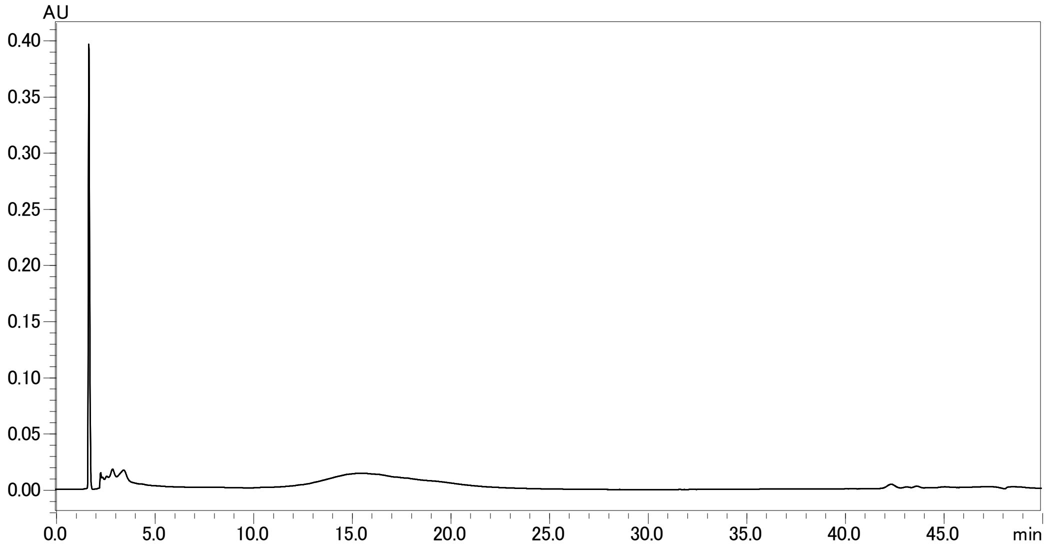

Figure 26. HPLC chromatogram (UV 280 nm) of the extract 21.

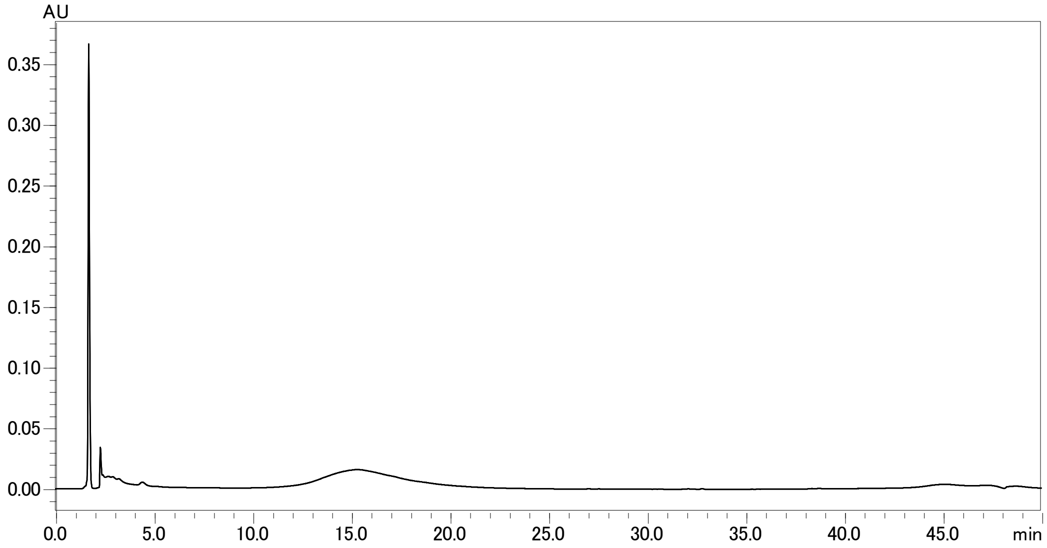

Figure 27. HPLC chromatogram (UV 280 nm) of the extract 22A.

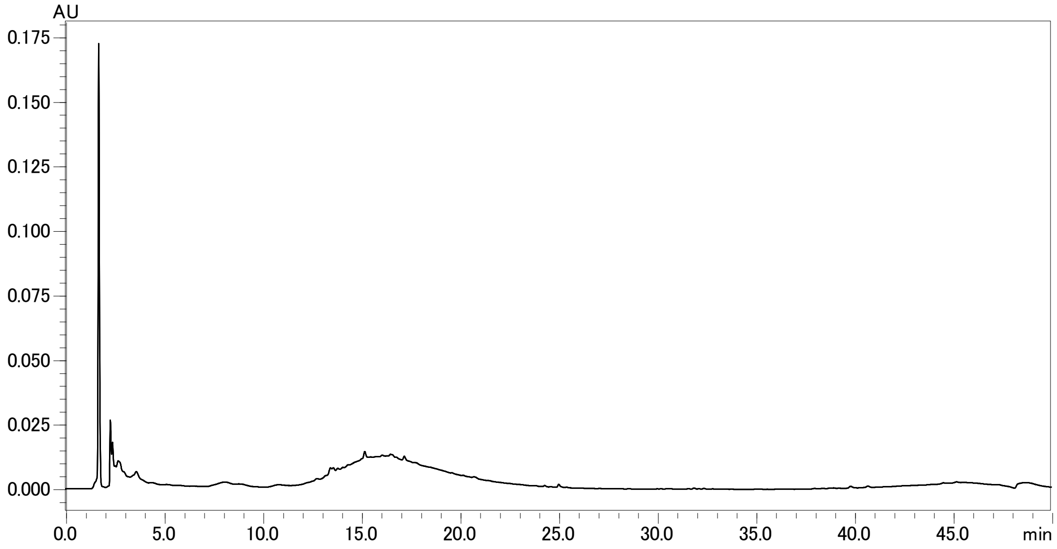

Figure 28. HPLC chromatogram (UV 280 nm) of the extract 22B.

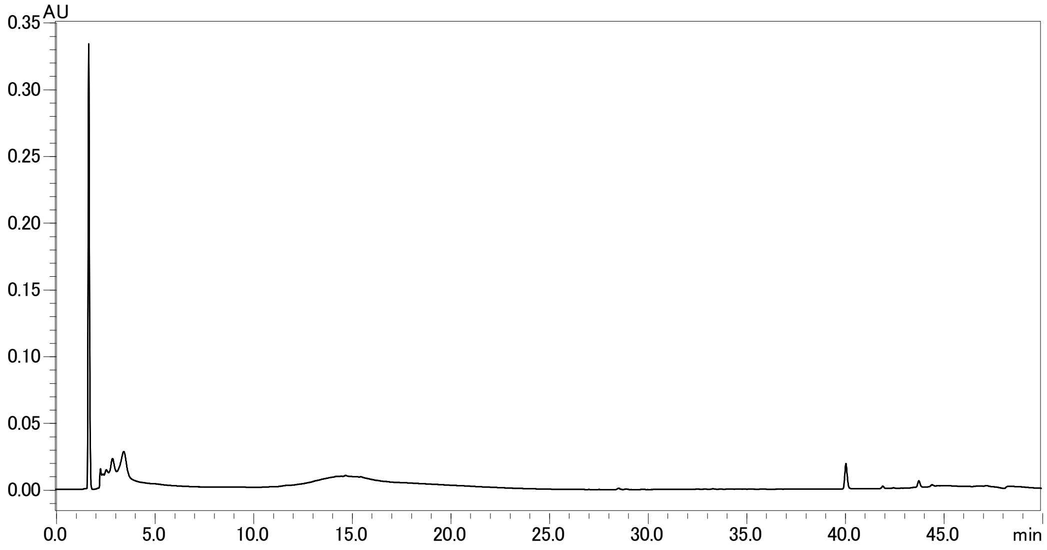

Figure 29. HPLC chromatogram (UV 280 nm) of the extract 23.

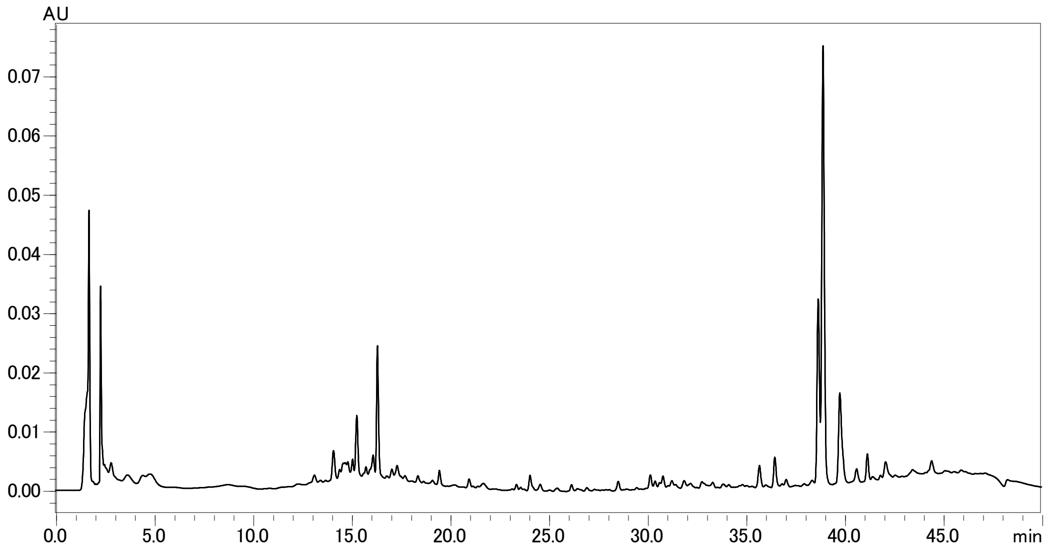

Figure 30. HPLC chromatogram (UV 280 nm) of the extract 24.

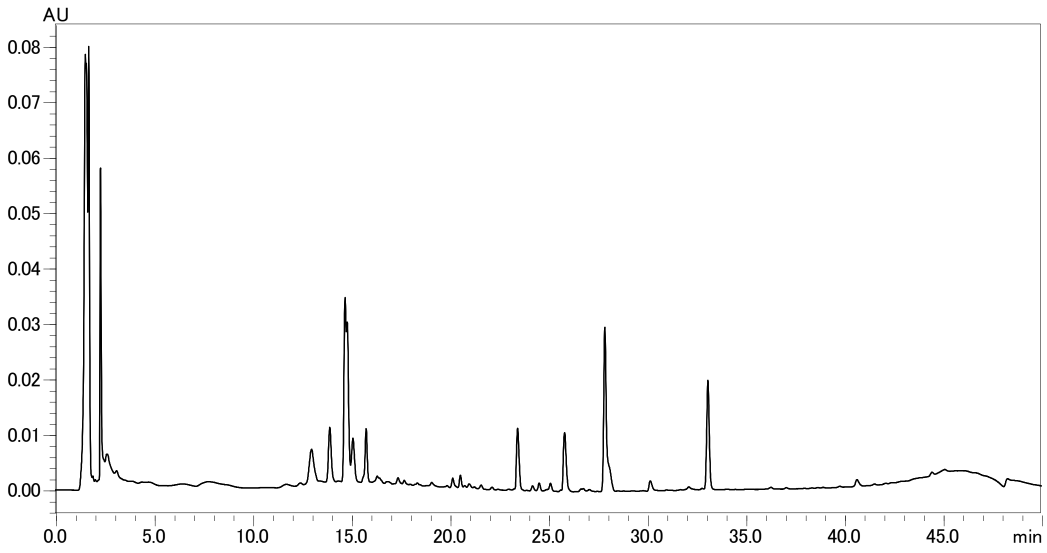

Figure 31. HPLC chromatogram (UV 280 nm) of the extract 25.

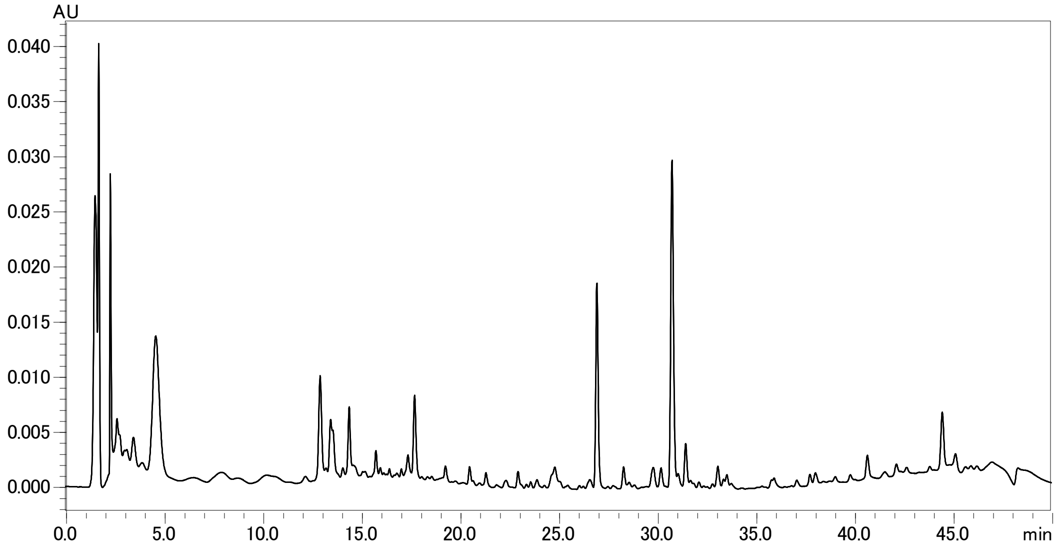

Figure 32. HPLC chromatogram (UV 280 nm) of the extract 26.

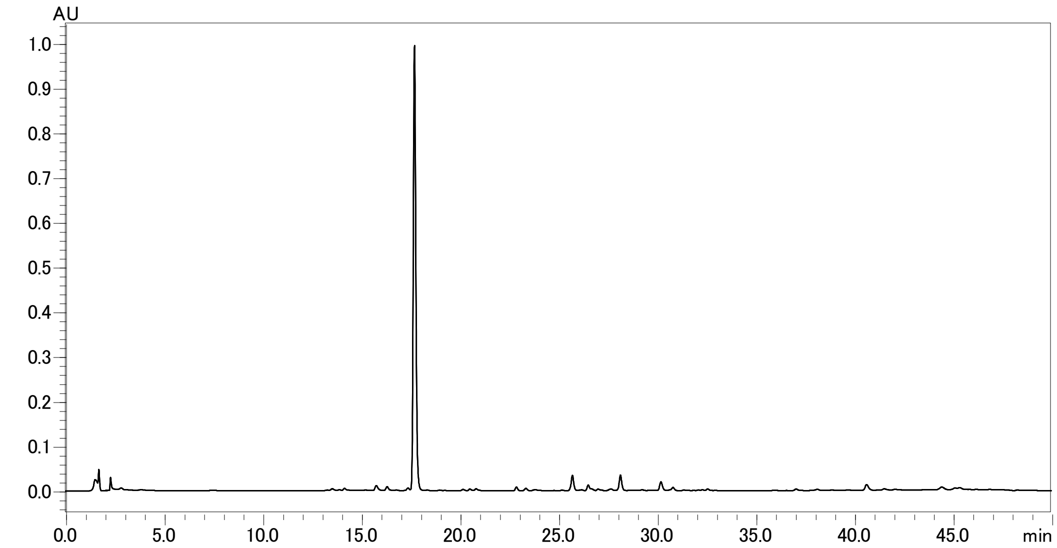

Figure 33. HPLC chromatogram (UV 280 nm) of the extract 27.

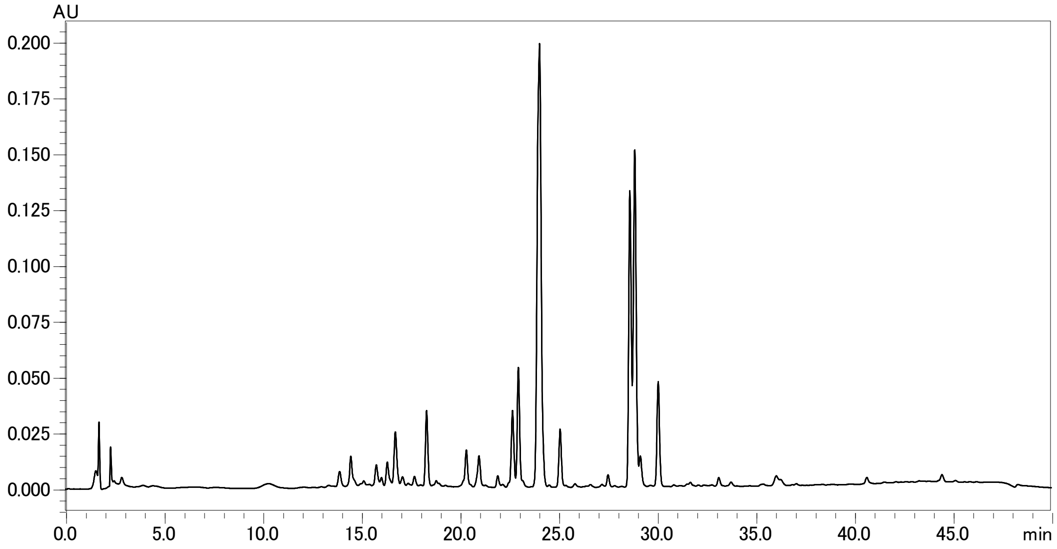

Figure 34. HPLC chromatogram (UV 280 nm) of the extract 28.

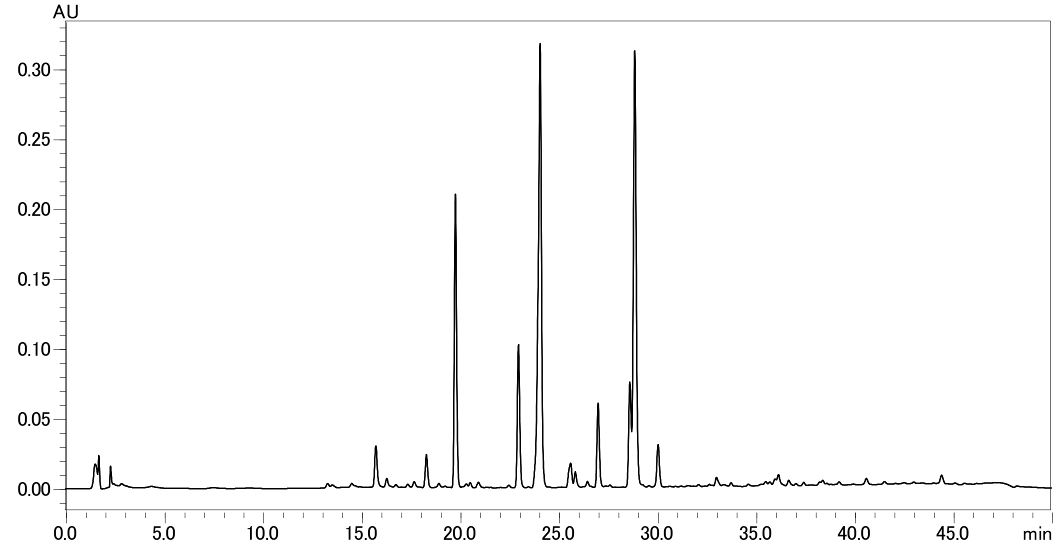

Figure 35. HPLC chromatogram (UV 280 nm) of the extract 29.

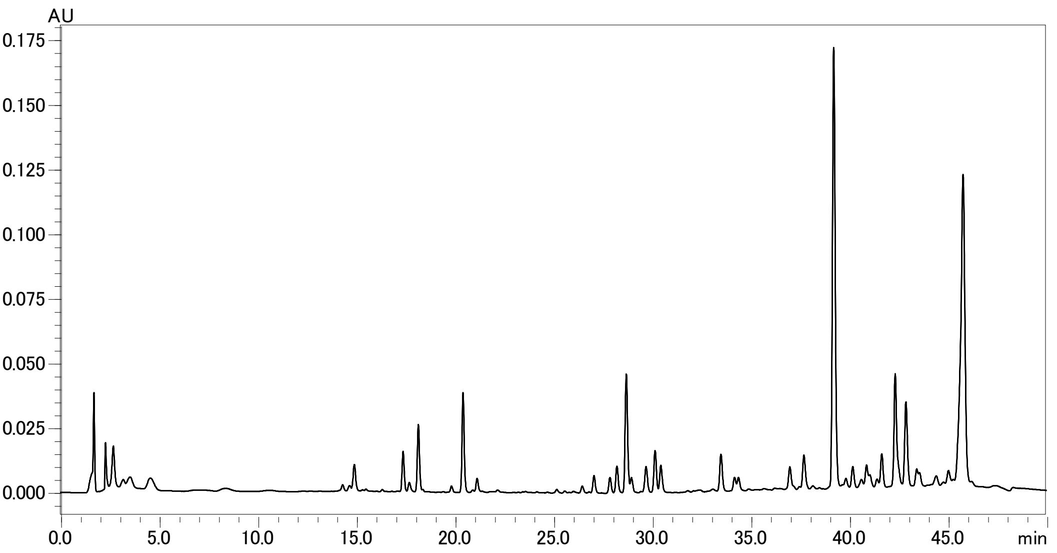

Figure 36. HPLC chromatogram (UV 280 nm) of the extract 30.

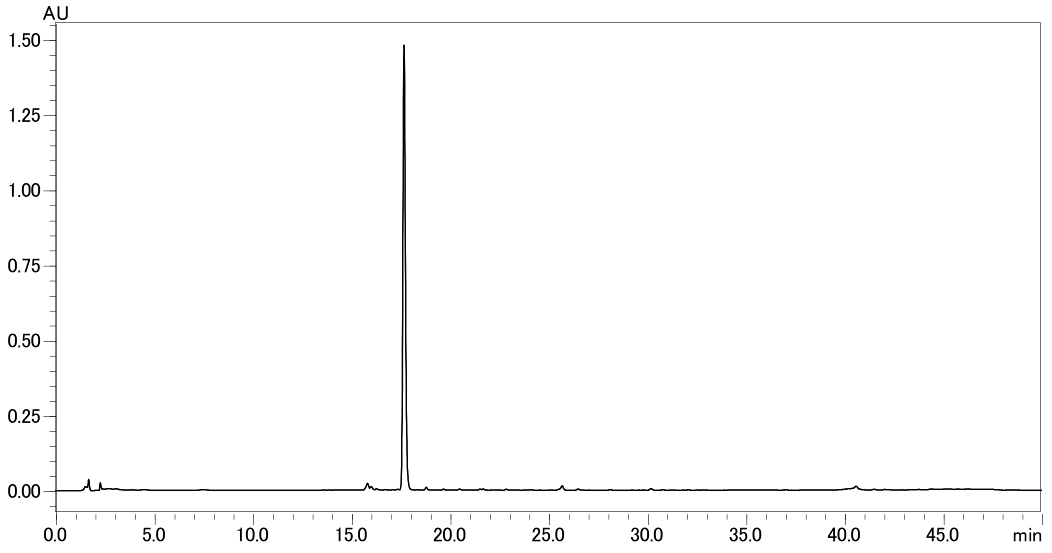

Figure 37. HPLC chromatogram (UV 280 nm) of the extract 31.

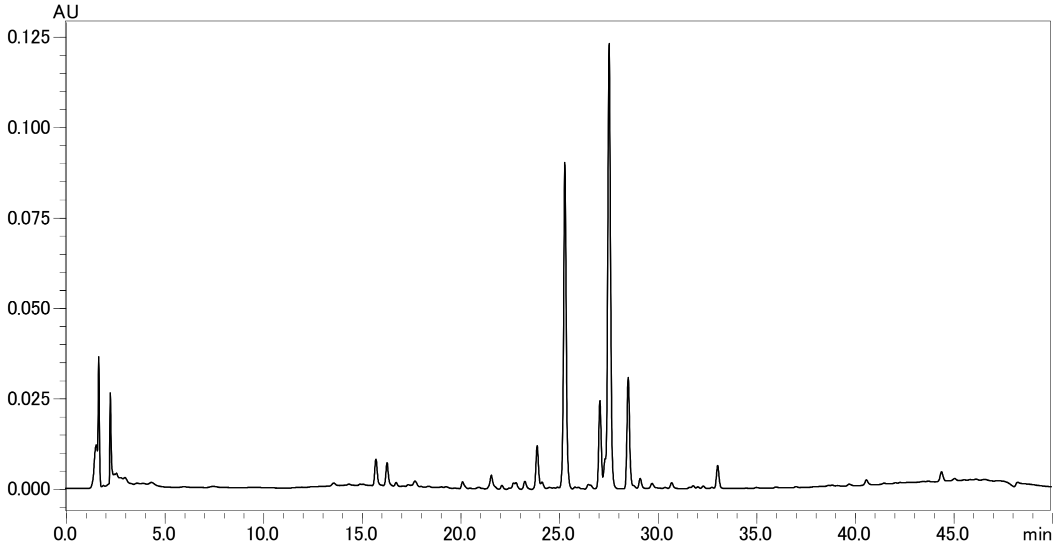

Figure 38. HPLC chromatogram (UV 280 nm) of the extract 32.

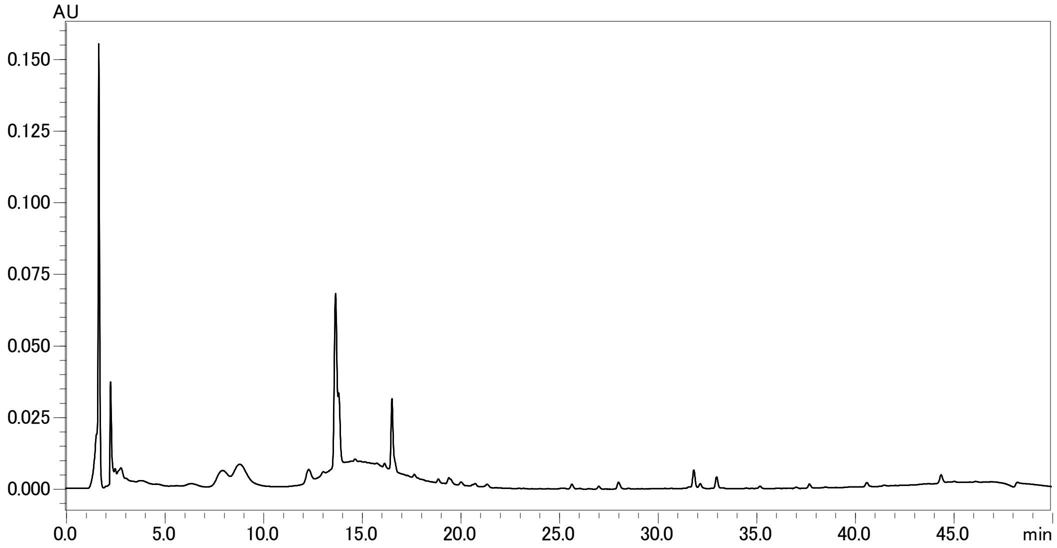

Figure 39. HPLC chromatogram (UV 280 nm) of the extract 33A.

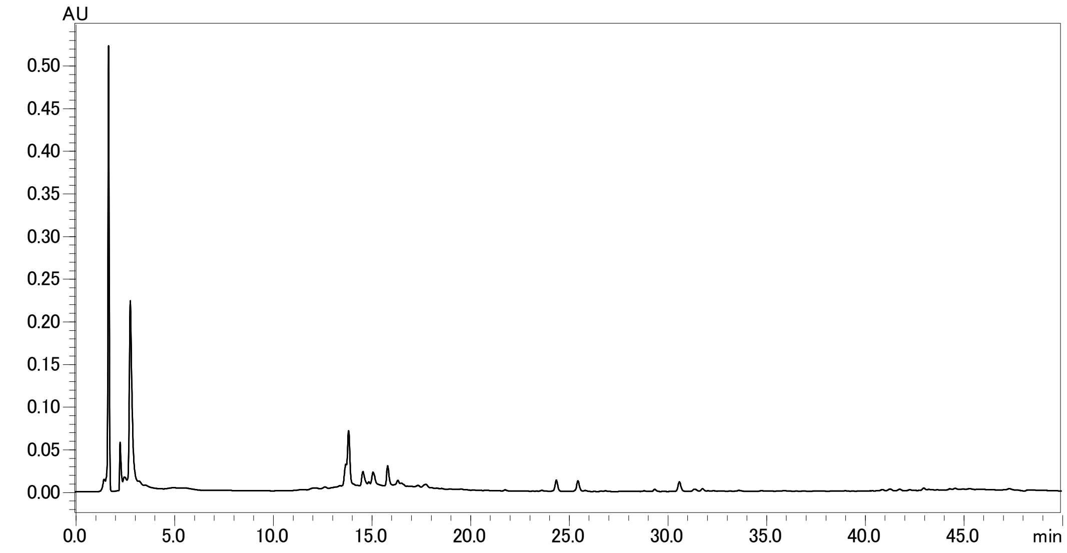

Figure 40. HPLC chromatogram (UV 280 nm) of the extract 33B.

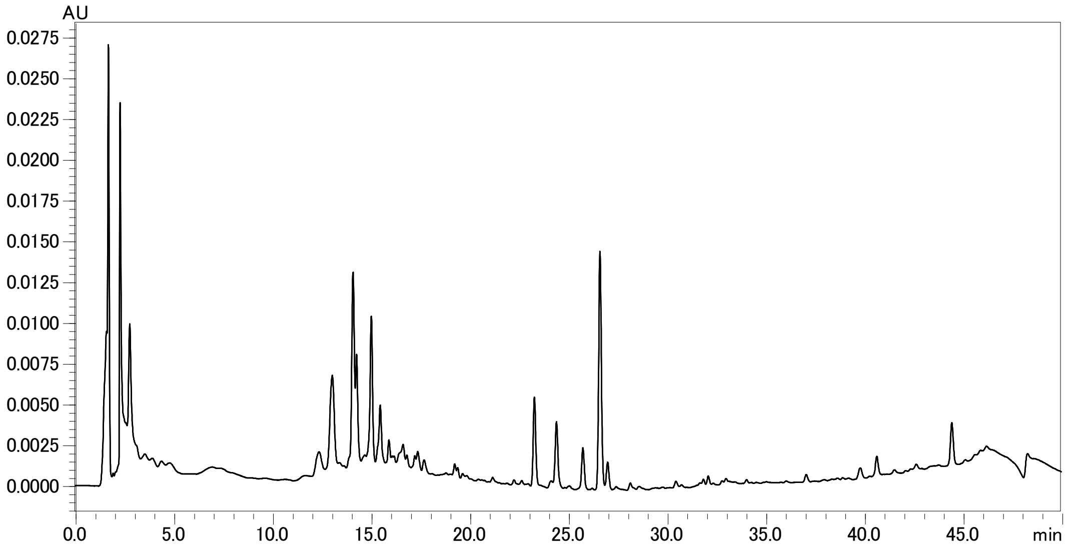

Figure 41. HPLC chromatogram (UV 280 nm) of the extract 34.

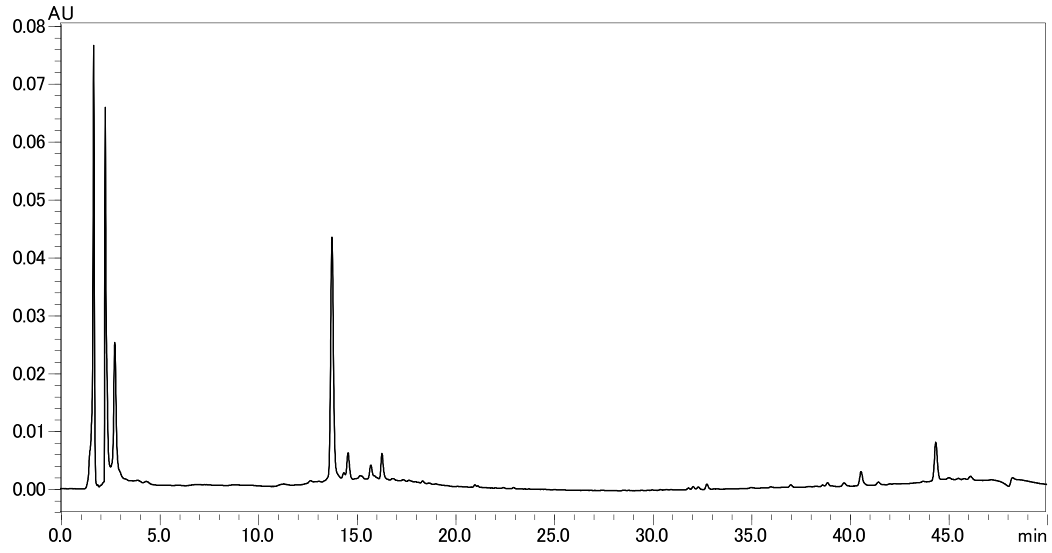

Figure 42. HPLC chromatogram (UV 280 nm) of the extract 35.

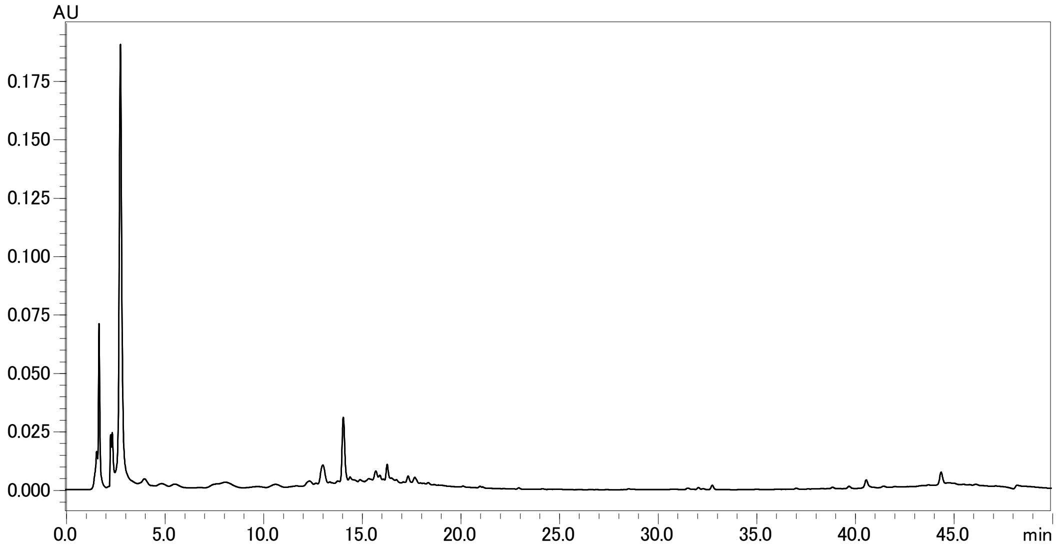

Figure 43. HPLC chromatogram (UV 280 nm) of the extract 36.

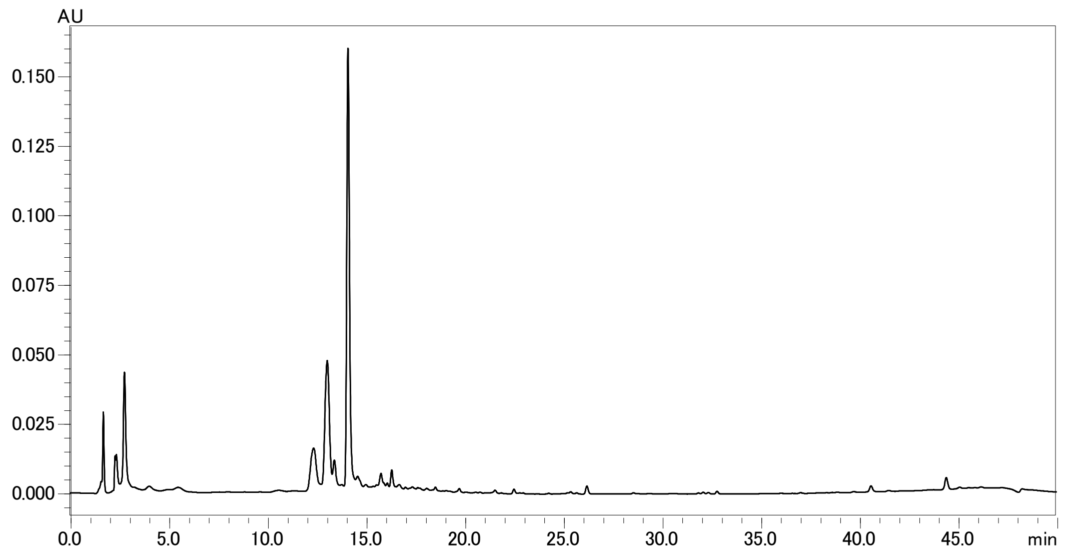

Figure 44. HPLC chromatogram (UV 280 nm) of the extract 37.

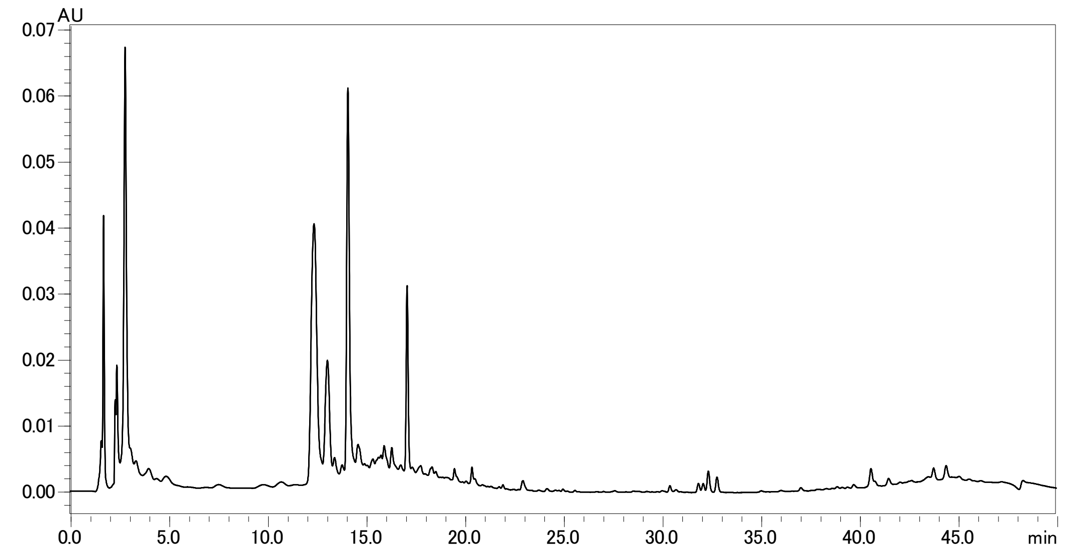

Figure 45. HPLC chromatogram (UV 280 nm) of the extract 38.

S25. Spectral data of known compounds

2,2-Dimethyl-6-carbomethoxychroman-4-one (**1**): white needles (from hexane-EtOH), mp 98–100°C (lit. 102°C); ESI-TOFMS (neg.) *m/z* 233.1 [M – H]$^-$; ^{1}H NMR (500 MHz, $CDCl_3$) δ: 8.55 (1H, d, *J* = 2.2 Hz, H-5), 8.13 (1H, dd, *J* = 8.7 Hz, 2.2 Hz, H-7), 6.97 (1H, d, *J* = 8.7 Hz, H-8), 3.90 (3H, s, OCH_3), 2.76 (2H, s, H-3), 1.49 (6H, s, H-2', H-3'). ^{13}C NMR (125 MHz, $CDCl_3$) δ: 191.5 (C-4), 166.3 (C-1'), 163.2 (C-9), 136.8 (C-7), 129.0 (C-5), 122.9 (C-10), 119.6 (C-6), 118.6 (C-8), 80.2 (C-2), 52.1 (OCH_3), 48.6 (C-3), 26.6 (C-1'' and C-2'').

2,2-Dimethyl-6-carboxychroman-4-one (**2**): white powder (from hexane-EtOH), mp 224.5–226.5°C (lit. 223–225°C); ESI-TOFMS (neg.) *m/z* 219.0 [M – H]$^-$; ^{1}H NMR (500 MHz, $CDCl_3$) δ: 8.65 (1H, d, *J* = 2.0 Hz, H-5), 8.18 (1H, dd, *J* = 8.7 Hz, 2.0 Hz, H-7), 7.01 (1H, d, *J* = 8.7 Hz, H-8), 2.78 (2H, s, H-3), 1.50 (6H, s, H-1', H-2'). ^{13}C NMR (125 MHz, $CDCl_3$) δ: 191.4 (C-4), 170.7 (C-1'), 163.8 (C-9), 137.3 (C-7), 130.0 (C-5), 122.0 (C-10), 119.7 (C-6), 118.8 (C-8), 80.4 (C-2), 48.6 (C-3), 26.6 (C-1'' and C-2'').

Cardamomin (**3**): yellow powder (from MeOH), mp 197.5–199.5℃ (lit. 195.5–196.5℃); ESI-TOFMS (neg.) *m/z* 269.1 [M – H]$^-$; ^{1}H NMR (500 MHz, acetone-*d*$_6$) δ: 14.15 (1H, s, OH), 8.02 (1H, d, *J* = 15.6 Hz, H-3), 7.69 (1H, d, *J* = 15.7 Hz, H-2), 7.63-7.65 (2H, m), 7.40-7.42 (3H, m), 6.01 (1H, d, *J* = 2.3 Hz, H-5'), 5.93 (1H, d, *J* = 2.3 Hz, H-3'), 3.93 (3H, s, OCH_3). ^{13}C NMR (125 MHz, acetone-*d*$_6$) δ: 193.0 (C-9), 168.3 (C-2'), 165.8 (C-4'), 164.3 (C-6'), 142.4 (C-8), 136.5 (C-1), 130.7 (C-4), 129.7 (C-3 and C-5), 129.0 (C-2 and C-6), 128.6 (C-7), 106.4 (C-1'), 97.0 (C-5'), 92.3 (C-3'), 56.3 (OCH_3).

Pinocembrin (**5**): white powder, mp 195–197°C (lit. 189–192°C); $[\alpha]_D^{25°C}$ +6.7 (c=0.001, MeOH) [lit. –58.5 (c=0.98, MeOH)]; ESI-TOFMS (neg.) *m/z* 269.1 [M – H]$^-$; ^{1}H NMR (500 MHz, MeOH-*d*$_4$) δ: 7.51 (2H, m, H-2'), 7.40 (2H, m, H-3'), 7.35 (1H, m, H-4'), 5.93 (1H, d, *J* = 2.2 Hz, H-8), 5.89 (1H, d, *J* = 2.2 Hz, H-6), 5.47 (1H, dd, *J* = 3.1 Hz, 12.7 Hz, H-2), 3.11 (1H, dd, *J* = 12.7 Hz, 17.1 Hz, H-3α), 2.79 (1H, dd, *J* = 3.1 Hz, 17.1 Hz, H-3β). ^{13}C NMR (125 MHz, MeOH-*d*$_4$) δ: 197.3 (C-4),

168.6 (C-7), 165.5 (C-5), 164.7 (C-9), 140.5 (C-1’), 129.7 (C-3’ and C-5’), 129.6 (C-4’), 127.4 (C-2’ and C-6’), 103.4 (C-10), 97.2 (C-6), 96.3 (C-8), 80.5 (C-2), 44.2 (C-3).

S27. NMR spectra of compound **4** ($CDCl_3$)

^{1}H-NMR spectrum of compound **4**

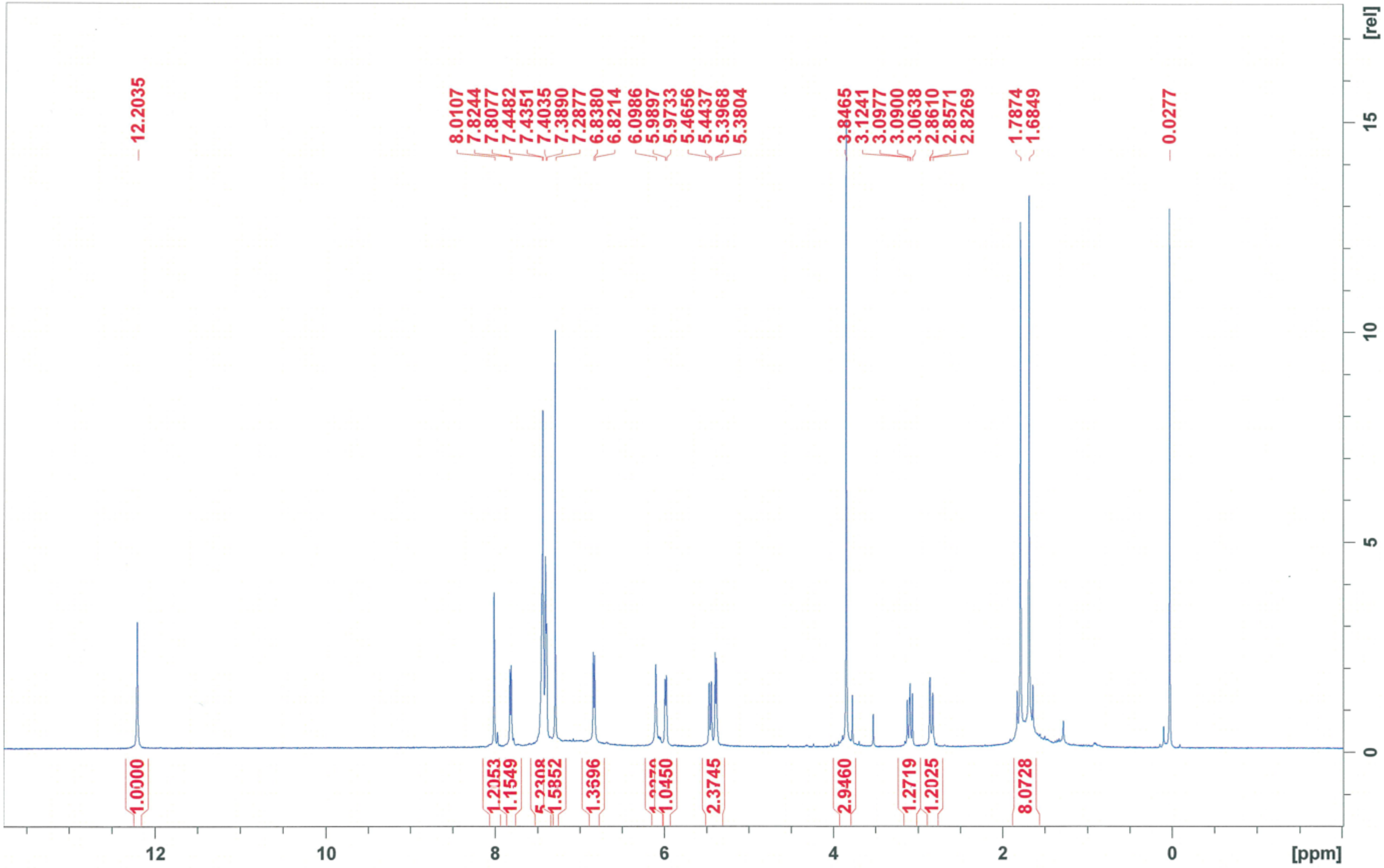

^{13}C-NMR spectrum of compound **4**

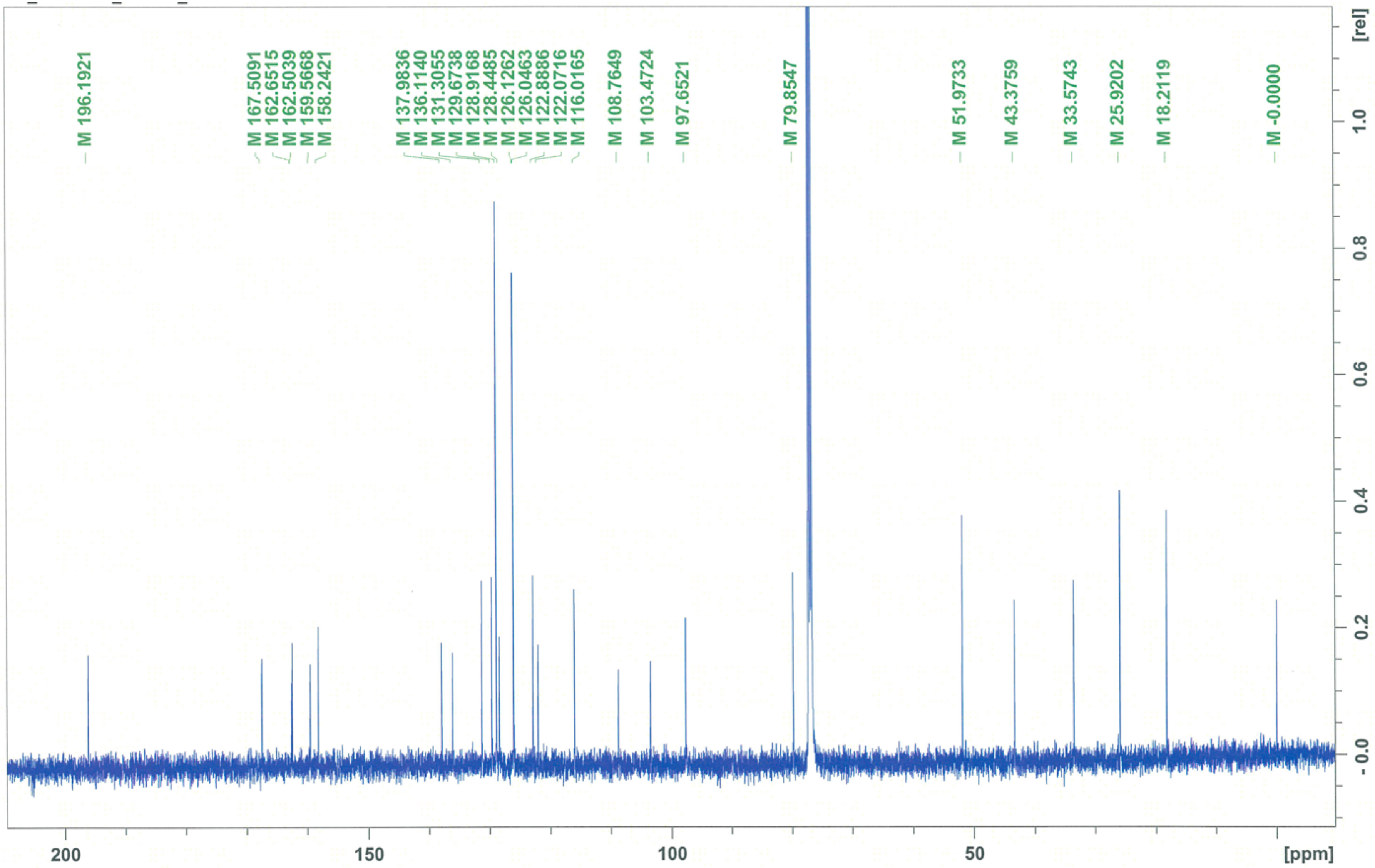

HMQC spectrum of compound **4**

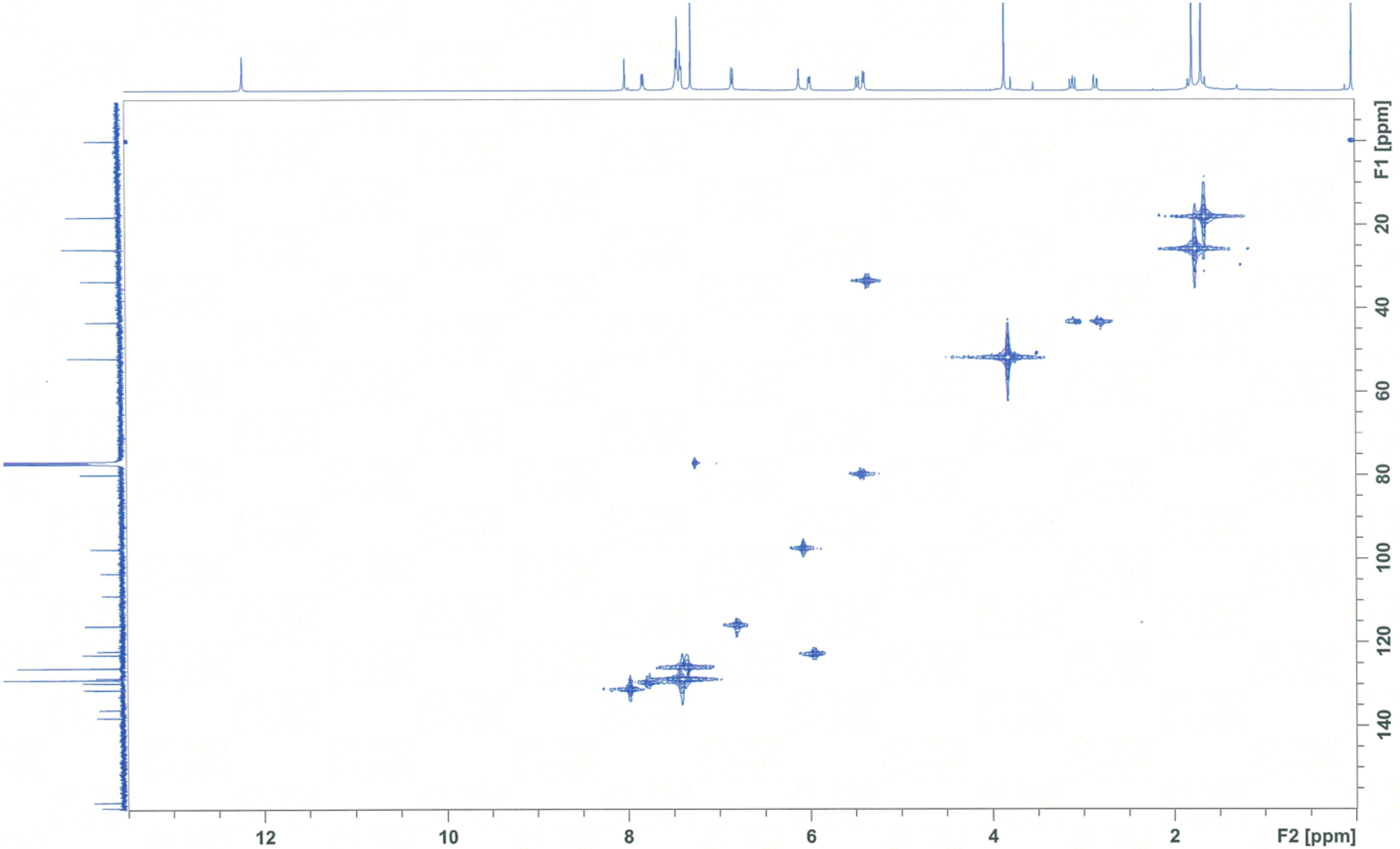

HMBC spectrum of compound **4**

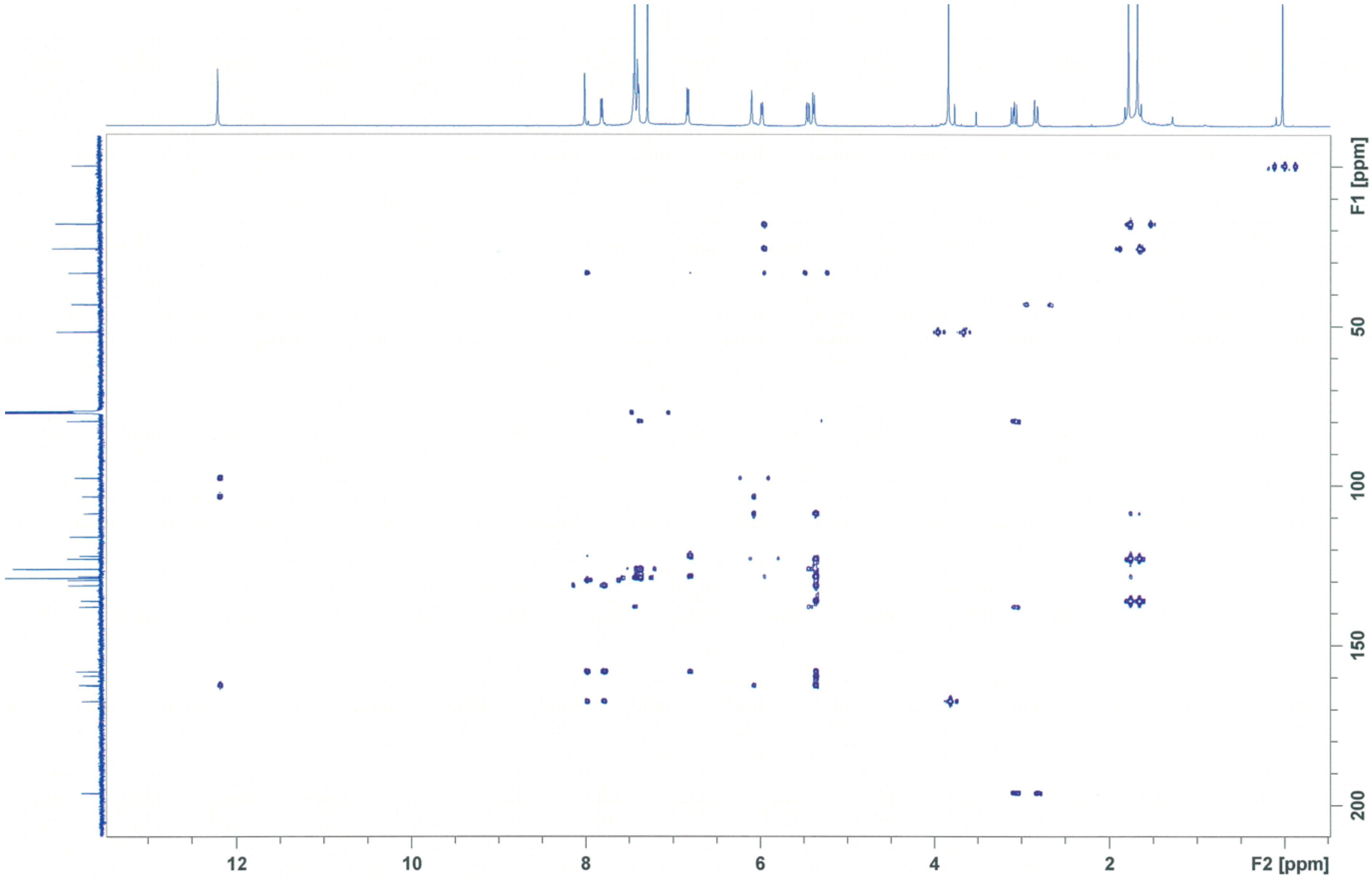

Supplement: Supplementary file 1 — Supplementary file1 (PDF 3565 kb) [file 11418_2021_1562_MOESM1_ESM.pdf]
